# Sodium-glucose cotransporter–2 (SGLT2) inhibitors and the reporting of falls and fractures: an european pharmacovigilance analysis

**DOI:** 10.3389/fphar.2023.1245642

**Published:** 2023-11-06

**Authors:** Annamaria Mascolo, Concetta Rafaniello, Gabriella di Mauro, Donatella Ruggiero, Maria Rosaria Campitiello, Maria Donniacuo, Pasquale Maria Berrino, Francesco Rossi, Giuseppe Paolisso, Annalisa Capuano

**Affiliations:** ^1^ Campania Regional Centre for Pharmacovigilance and Pharmacoepidemiology, Naples, Italy; ^2^ Department of Experimental Medicine–Section of Pharmacology “L. Donatelli”, University of Campania “Luigi Vanvitelli”, Naples, Italy; ^3^ UOC Pharmacy, AORN Santobono-Pausilipon Children’s Hospital, Naples, Italy; ^4^ Department of obstetrics and gynaecology and physiopathology of human reproduction, ASL Salerno, Salerno, Italy; ^5^ Department of Specialized Medicine, Diagnostic and Experimental, University of Bologna “Alma Mater Studiorum”, Bologna, Italy; ^6^ Department of Advanced Medical and Surgical Sciences, University of Campania “Luigi Vanvitelli”, Naples, Italy; ^7^ UniCamillus, International Medical University, Rome, Italy

**Keywords:** SGLT2 inhibitors, DPP4 inhibitors, fall, fracture, safety

## Abstract

**Background:** The risk of falls and bone fractures with sodium-glucose co-transporter-2 (SGLT2) inhibitors has been characterized by conflicting evidence. Therefore, we decided to investigate the reporting probability of falls and fractures by comparing SGLT2 inhibitors with DPP4 inhibitors.

**Methods** A retrospective, pharmacovigilance study of the European database of Individual Case Safety Reports (ICSRs) was conducted. Disproportionality analyses (Reporting Odds Ratio, ROR) were conducted to compare the reporting probability of falls or fracture between treatments.

**Results** A total of 507 ICSRs reporting at least one fall or fracture with SGLT2 inhibitors were identified. The most reported SGLT2 inhibitor was canagliflozin (*N* = 188; 36.9%), followed by empagliflozin (*N* = 176; 34.5%), and dapagliflozin (*N* = 143; 28.0%). A total of 653 events related to fall or bone fracture were reported. Fall was the most reported event (*N* = 333; 51.0%). Among fractures (*N* = 320; 49.0%), the most reported were foot fractures (*N* = 40; 6.1%) and hip fractures (*N* = 32; 4.9%). SGLT2 inhibitors were associated with a lower reporting probability of fall than DPP4 inhibitors (ROR, 0.66; 95%CI, 0.57-0.78). The lower reporting probability of fall was also observed when the single SGLT2 inhibitor was compared to DPP4 inhibitors: dapagliflozin (ROR, 0.67; 95%CI, 0.53-0.83), canagliflozin (ROR, 0.56; 95%CI, 0.45-0.70), and empagliflozin (ROR, 0.77; 95%CI, 0.63-0.94). For fractures, canagliflozin showed a slightly significant increased reporting when compared with DPP4 inhibitors (not confirmed in the sensitivity analysis), whereas all other comparison showed no statistically significant difference.

**Conclusion** SGLT2 inhibitors were associated with a lower reporting probability of fall than DPP4 inhibitors, in accordance with the reassuring evidence about the safety profile of these drugs. Future researches will help to confirm their long-term safety profile.

## 1 Introduction

Sodium-glucose co-transporter-2 (SGLT2) inhibitors were firstly authorized for the treatment of type 2 diabetes mellitus, but subsequently dapagliflozin and empagliflozin also for the treatment of heart failure with reduced ejection fraction based on their favorable cardiovascular outcomes (Mascolo et al., 2022b). Indeed, SGLT2 inhibitors have also shown pleiotropic effects able to determine cardiovascular and renal protection besides the hypoglycemic effect (Mascolo et al., 2021; Mascolo et al., 2022a; Mascolo et al., 2022b).

Despite their clinical benefits, these drugs have also been associated with safety concerns in different cardiovascular trials, some of which have already been implemented in the summary of product’s information by Regulatory Agencies (Mascolo et al., 2022b). Among safety warnings, the risk of fractures and falls, which can sometime be related with each other, has been characterized by conflicting evidence (Ye et al., 2018). Specifically, canagliflozin was firstly associated with an increased risk of bone fractures in the Canagliflozin Cardiovascular Assessment Study (CANVAS) (Neal et al., 2017). However, this risk was not confirmed in other large randomized clinical trials (RCTs) of canagliflozin or other SGLT2 inhibitors (Zinman et al., 2015; McMurray et al., 2019; Perkovic et al., 2019; Wiviott et al., 2019; Heerspink et al., 2020). Moreover, no difference in the risk of fractures between SGLT2 inhibitors and dipeptidyl peptidase-4 (DPP4) inhibitors or glucagon-like peptide 1 (GLP-1) receptor agonists was found in a population-based cohort study conducted on US data related to older adults (Zhuo et al., 2021). Another observational, retrospective, pharmacovigilance study conducted on data from the US Food and Drug Administration Adverse Event Reporting System (FAERS) showed no association between SGLT2 inhibitors alone or combined with other glucose-lowering medications and the risk of fractures (Zhao et al., 2021). The risk of falls with SGLT2 inhibitors has instead been less investigated. The US cohort study found a lower risk of falls when SGLT2 inhibitors were compared with DPP4 inhibitors, while no difference was observed when compared with GLP-1 receptor agonists (Zhuo et al., 2021). Another pharmacovigilance safety study conducted only on FAERS data also found no difference for fall when SGLT-2 inhibitors were compered to other non-insulin antidiabetic agents (Goldman et al., 2023). Generally, the evidence suggested that the increased rates of neuropathy and orthostatic hypotension observed with SGLT2 inhibitors can facilitate the onset of accidental falls and then fractures (Kohan et al., 2014). However, a prospective multicenter study found that SGLT2 inhibitors might also significantly modulate the cardiac autonomic neuropathy dysfunction in type 2 diabetes mellitus patients with vasovagal syncope leading to a reduction of vasovagal syncope recurrence in these patients (Sardu et al., 2022). For DPP4 inhibitors, a study found a higher reporting of events related to the gastrointestinal tract, pancreas, malignancies, infection, musculoskeletal disorders, general disorders, hypersensitivity, and skin reactions, but no safety signal was observed for fractures events (Huang et al., 2020). Moreover, the SAVOR-TIMI 53 trial did not find any difference between the DPP4 inhibitor saxagliptin and placebo (Mosenzon et al., 2015), while a meta-analysis found a reduced risk with DPP4 inhibitors (Monami et al., 2011). Considering the conflicting and meagre evidence available, especially for the risk of falls, and the lack of European pharmacovigilance studies, we decided to conduct a Real-World safety study to compare Individual Case Safety Reports (ICSRs) of falls and bone fractures between SGLT2 inhibitors and DPP4 inhibitors by using the European Pharmacovigilance database (EudraVigilance, EV).

## 2 Methods

### 2.1 Study design

A retrospective, European, pharmacovigilance study aiming to compare the reporting probability of falls or bone fractures between SGLT2 inhibitors and DPP4 inhibitors.

### 2.2 Study period and data source

The EV was used to retrieve ICSRs from 1 January 2015 to 31 December 2022 for both SGLT2 and DPP4 inhibitors. Considering the different dates of marketing authorization, the study start date was chosen to make the post-authorization years equal among SGLT2 inhibitors and DPP4 inhibitors. Only ertugliflozin was authorized in Europe after the study start date (21 March 2018); therefore, it was excluded from analyses.

The EV is a database of European Medicines Agency (EMA) intended for the collection, management, and analysis of ICSRs related to medicines or vaccines authorized or under investigation in clinical trials in the European Economic Area (EEA). Healthcare professionals (HCP) or non-HCPs can spontaneously report an ICSR to a marketing authorization holder or an EU national competent authority, which are then responsible for their registration in EV. Data contained in EV are available at www.adrreports.eu.

### 2.3 ICSRs selection

ICSRs with SGLT2 inhibitors (dapagliflozin, canagliflozin, and empagliflozin) or DPP4 inhibitors (sitagliptin, saxagliptin, vildagliptin, alogliptin, and linagliptin) as suspected drugs were selected by using the line listing function of EV. DPP4 inhibitors were chosen as the comparator because they are oral drugs frequently used as second-line therapy for type 2 diabetes mellitus. Moreover, they seem to be neutral on the risk of falls and bone fractures (Hou et al., 2018). Selection criteria for cases were: the presence among suspected drugs of at least one active ingredient or medicinal product of an SGLT2 inhibitor or DPP4 inhibitor as monotherapy and the presence among adverse events of a fall or bone fracture. To identify fall or bone fractures, we used the preferred terms (PTs) of the System Organ Classes (SOCs) of the Medical Dictionary for Regulatory Activities (MedDRA) “Injury, poisoning and procedural complications” of MedDRA version 24.1. Specifically, the PT “fall” and all PTs containing the word “fracture” and indicative of a broken bone in any part of the body were used for selection (Supplementary Table S1). Since we wanted to evaluate the effect of single SGLT2 inhibitor products, all ICSRs available as combination medicinal products such as an SGLT2 inhibitor plus metformin or SGLT2 inhibitor plus DPP4 inhibitor were excluded. ICSRs reporting more than one SGLT2 inhibitor as monotherapy among suspected drugs were grouped as “combination”. After downloading datasets for each SGLT2 inhibitor, we merged them into one dataset that was checked for duplication removal based on the EV code and information on patients’ characteristics and events.

### 2.4 Study endpoints

The primary study endpoint was the reporting probability of fall of SGLT2 inhibitors compared to DPP4 inhibitors. Secondarily, since sometimes bone fractures can be associated with falls or *vice versa*, and considering that in the spontaneous reporting it can be reported only one of these events due to quality biases, we also assess the reporting probability of bone fractures (intended as the sum of all events of fractures) of SGLT2 inhibitors compared to DPP4 inhibitors.

### 2.5 Descriptive analyses

For descriptive purposes, the following information from ICSRs with SGLT2 inhibitors or DPP4 inhibitors as suspected drugs were retrieved: patient’s characteristics (age group and sex), type of reporter (HCP or non-HCP), primary Source Country for regulatory purposes (EEA or non-EEA), number of reported suspected drugs, and concomitant drugs. Considering that SGLT2 inhibitors can be used to treat two different diseases (diabetes mellitus and heart failure), we tabled their therapeutic indications as High-Level Group Term (HLGT) of the MedDRA. Events of fall and fracture related to SGLT2 inhibitors were tabled for suspected drugs and described in terms of type of event, outcome, and seriousness. According to the International Council on Harmonization E2D guidelines, an event was classified as serious if it met the following criteria: results in death, hospitalization or its prolongation, severe or permanent disability, congenital abnormalities/birth defects, life-threatening, or a clinically relevant condition. The outcome of the event was classified into: “recovered/resolved”, “recovering/resolving”, “recovered/resolved with sequelae”, “not recovered/not resolved”, “fatal”, or “unknown”. The median time to onset (TTO) of fall and fractures and its interquartile range (IQR), expressed in days, were also computed when the information on the duration of therapy and drug discontinuation as action taken was available in the ICSR.

To assess cardiovascular risk factors for fall, ICSRs were also described for the presence among therapeutic indications (where available) of other suspected or concomitant drugs of atrial fibrillation, hypotension, dizziness, or hypoglycemia; or the presence among other suspected or concomitant drugs of cardiovascular treatments [such as beta-blockers, angiotensin receptor blockers, angiotensin converting enzyme (ACE) inhibitors, antiplatelet, anticoagulants, etc.,].

### 2.6 Disproportionality analyses

The Reporting Odds Ratio (ROR), its 95% Confidence interval (95%CI), and the chi-square test were computed to compare the reporting probability of fall and fractures between SGLT2 inhibitors and DPP4 inhibitors. The ROR was calculated as (a/c)/(b/d): “a” is the number fall or fractures reported with the SGLT2 inhibitor, “c” the number of fall or fractures with the comparator (DPP4 inhibitors), “b” the number of other events reported with the SGLT2 inhibitor, and “d” the number of other events reported with comparator. This analysis was also computed to evaluate the reporting probability of fall and fractures between SGLT2 inhibitors and DPP4 inhibitors in only male or female, and in only cases aged ≥65 years or <65 years. Moreover, this analysis was performed to assess the reporting probability of both events comparing sex (female vs male) and age (≥65 years vs. < 65 years) within the class of SGLT2 inhibitors. To evaluate the impact of cardiovascular risk factors on the reporting probability of fall, a sensitivity analysis was performed by comparing cases without predisposing risk factors to cases with risk factors for each SGLT2 inhibitor. Finally, considering that an ICSR could report both treatments (SGLT2 inhibitors and DPP4 inhibitors), we performed a sensitivity analysis by excluding such ICSRs. All analyses were performed only for events reported at least 3 times for each drug. A 5% significance level was considered for all analyses. RORs were displayed with forest plots. Data management and analyses were performed with R (version 4.2.2, R Development Core Team).

## 3 Results

**FIGURE 1:**
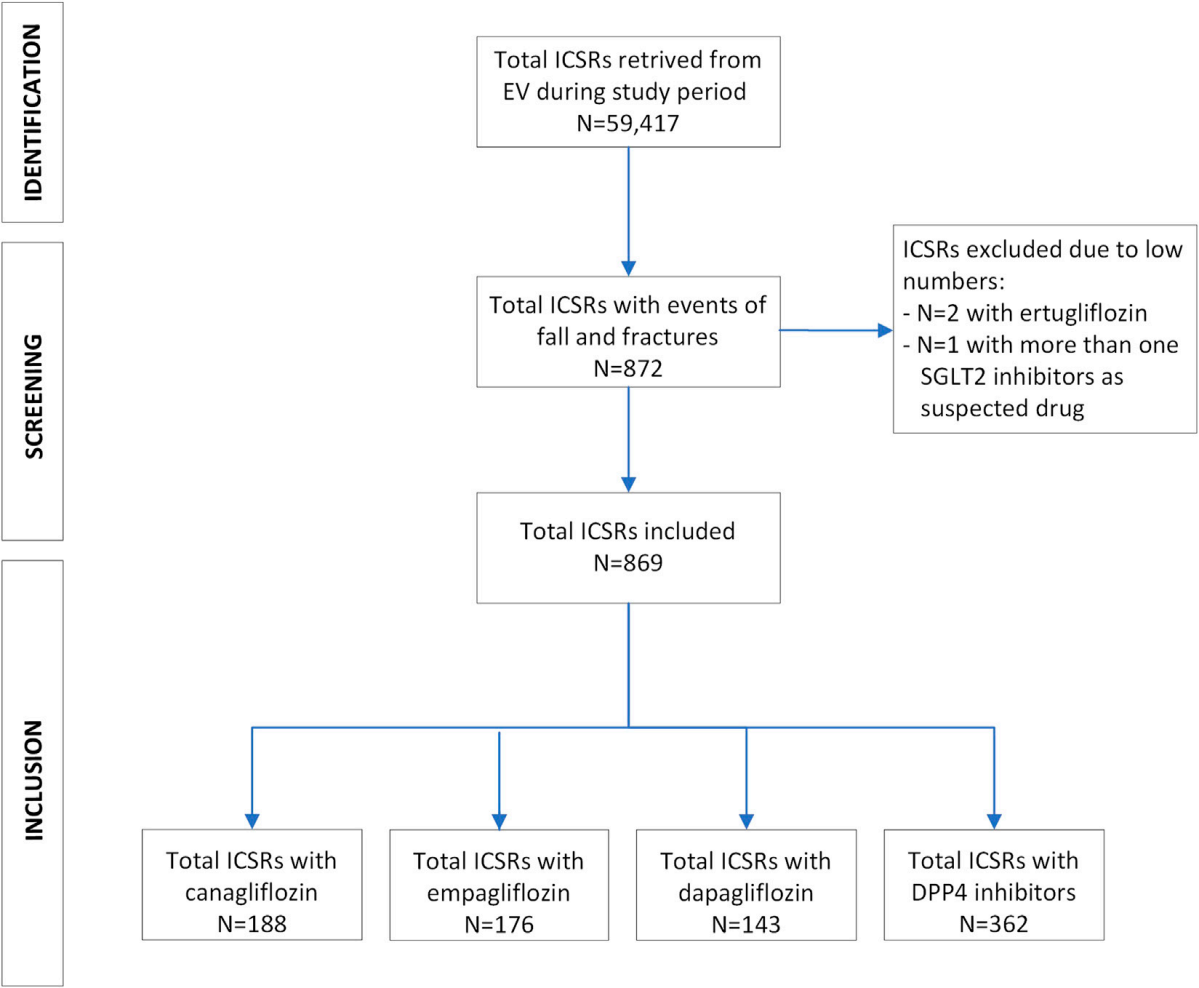
Selection process of cases of fall and fractures with SGLT2 inhibitors and DPP4 inhibitors during study period. DPP4: Dipeptidyl peptidase 4; EV: EudraVigilance; ICSRs: Individual Case Safety Reports; SGLT2: Sodium-glucose cotransporter 2.

During the study period, a total of 507 ICSRs reported at least one event of fall or fractures with SGLT2 inhibitors. The most reported SGLT2 inhibitor was canagliflozin (N = 188; 36.9%), followed by empagliflozin (N = 176; 34.5%), and dapagliflozin (N = 143; 28.0%). Only 1 (0.2%) ICSR reported the combination of two SGLT2 inhibitors (dapagliflozin and empagliflozin) and it was excluded from the analyses due to the low number. Most ICSRs were related to patients aged 65–85 years (N = 223; 44.0%) and mainly reported in females (N = 291; 57.4%). Most ICSRs were reported by HCPs (N = 319; 62.9%), and came from the non-EEA (N = 447; 88.2%). The SGLT2 inhibitor was the only suspected drug reported (N = 385; 75.9%) and no concomitant drug (N = 226; 44.6%) was reported in most ICSRs. A total of 362 ICSRs reported at least one event of fall and fractures, with sitagliptin as the most reported DPP4 inhibitors (N = 211; 58.3%), followed by linagliptin (N = 74; 20.4%), vildagliptin (N = 46; 12.7%), saxagliptin (N = 17; 4.7%), and alogliptin (N = 12; 3.3%). Only two ICSRs (0.5%) reported both sitagliptin and saxagliptin as suspected drugs. Characteristics of ICSRs for each SGLT2 inhibitor and for DPP4 inhibitors were reported in Table 1. A flowchart of the data selection process was reported in Figure 1. Therapeutic indications were shown in Table 1.

**TABLE 1:** Distribution for age group, sex, type of reporter, primary source country for regulatory purposes, number of suspected or concomitant drugs, and therapeutic indications among Individual Case Safety Reports (ICSRs) reporting a fall or fracture with an SGLT2 inhibitor and reported in Eudravigilance from January 1st. 2015 to December 31st.

	Canagliflozin (*N* = 188)	Empagliflozin (*N* = 176)	Dapagliflozin (*N* = 143)	All SGLT2 inhibitors (*N* = 507)	DPP4 inhibitors (*N* = 362)
Age group					
*18–64* *Years*	85 (45.2%)	39 (22.2%)	38 (26.6%)	162 (32.0%)	64 (17.7%)
*65–85* *Years*	69 (36.7%)	90 (51.1%)	64 (44.8%)	223 (44.0%)	163 (45.0%)
*More than 85* *Years*	8 (4.3%)	3 (1.7%)	9 (6.3%)	20 (3.9%)	43 (11.9%)
*Not Specified*	26 (13.8%)	44 (25.0%)	32 (22.4%)	102 (20.1%)	92 (25.4%)
Sex					
*Female*	106 (56.4%)	104 (59.1%)	81 (56.6%)	291 (57.4%)	216 (59.7%)
*Male*	78 (41.5%)	63 (35.8%)	57 (39.9%)	198 (39.0%)	139 (38.4%)
*Not Specified*	4 (2.1%)	9 (5.1%)	5 (3.5%)	18 (3.5%)	7 (1.9%)
Reporter					
*Healthcare Professional*	148 (78.7%)	96 (54.5%)	75 (52.4%)	319 (62.9%)	264 (72.9%)
*Non Healthcare Professional*	40 (21.3%)	80 (45.5%)	68 (47.6%)	188 (37.1%)	98 (27.1%)
Primary source country for regulatory purposes					
*European Economic Area*	3 (1.6%)	33 (18.8%)	24 (16.8%)	60 (11.8%)	37 (10.2%)
*Non European Economic Area*	185 (98.4%)	143 (81.3%)	119 (83.2%)	447 (88.2%)	325 (89.8%)
Concomitant drugs					
*0*	101 (53.7%)	80 (45.5%)	45 (31.5%)	226 (44.6%)	141 (39.0%)
*1*	11 (5.9%)	18 (10.2%)	20 (14.0%)	49 (9.7%)	36 (9.9%)
*2*	9 (4.8%)	10 (5.7%)	8 (5.6%)	27 (5.3%)	19 (5.2%)
*3*	7 (3.7%)	12 (6.8%)	9 (6.3%)	28 (5.5%)	26 (7.2%)
*4*	8 (4.3%)	6 (3.4%)	7 (4.9%)	21 (4.1%)	19 (5.2%)
*5*	52 (27.7%)	50 (28.4%)	54 (37.8%)	156 (30.8%)	121 (33.4%)
Suspected drugs					
*1*	156 (83.0%)	132 (75.0%)	95 (66.4%)	383 (75.5%)	144 (39.8%)
*2*	23 (12.2%)	28 (15.9%)	26 (18.2%)	77 (15.2%)	57 (15.7%)
*3*	2 (1.1%)	7 (4.0%)	11 (7.7%)	20 (3.9%)	34 (9.4%)
*4*	4 (2.1%)	4 (2.3%)	4 (2.8%)	12 (2.4%)	23 (6.4%)
*5*	3 (1.6%)	5 (2.8%)	7 (4.9%)	15 (3.0%)	104 (28.7%)
Therapeutic indication					
*Glucose metabolism disorders (incl diabetes mellitus)*	130 (69.1%)	102 (58.0%)	93 (65.0%)	325 (64.1%)	230 (63.5%)
*Metabolic, nutritional and blood gas investigations*	1 (0.5%)	1 (0.6%)	3 (2.1%)	4 (0.8%)	3 (0.8%)
*Unknown*	57 (30.3%)	60 (34.1%)	2 (1.4%)	119 (23.5%)	86 (23.8%)
*Not available*	0 (0%)	5 (2.8%)	27 (18.9%)	32 (6.3%)	40 (11.0%)
*Appetite and general nutritional disorders*	0 (0%)	0 (0%)	1 (0.7%)	1 (0.2%)	0 (0%)
*Cardiac and vascular investigations (excl enzyme tests)*	0 (0%)	0 (0%)	1 (0.7%)	1 (0.2%)	0 (0%)
*Heart failures*	0 (0%)	3 (1.7%)	8 (5.6%)	11 (2.2%)	0 (0%)
*Heart failures, Glucose metabolism disorders (incl diabetes mellitus)^a^*	0 (0%)	2 (1.1%)	3 (2.1%)	5 (1.0%)	0 (0%)
*Heart failures, Urinary tract signs and symptoms^a^*	0 (0%)	0 (0%)	1 (0.7%)	1 (0.2%)	0 (0%)
*Metabolic, nutritional and blood gas investigations, Glucose metabolism disorders (incl diabetes mellitus)^a^*	0 (0%)	0 (0%)	1 (0.7%)	1 (0.2%)	0 (0%)
*Renal disorders (excl nephropathies)*	0 (0%)	0 (0%)	3 (2.1%)	3 (0.6%)	1 (0.3%)
*General system disorders NEC*	0 (0%)	0 (0%)	0 (0%)	0 (0%)	1 (0.3%)
*Nervous system, skull and spine therapeutic procedures, Vascular hypertensive disorders^a^*	0 (0%)	0 (0%)	0 (0%)	0 (0%)	1 (0.3%)
*Cardiac disorders, signs and symptoms NEC*	0 (0%)	1 (0.6)	0 (0%)	1 (0.2%)	0 (0%)
*Cardiac disorders, signs and symptoms NEC, Glucose metabolism disorders (incl diabetes mellitus)^a^*	0 (0%)	1 (0.6)	0 (0%)	1 (0.2%)	0 (0%)
*Coronary artery disorders, Glucose metabolism disorders (incl diabetes mellitus)^a^*	0 (0%)	1 (0.6)	0 (0%)	1 (0.2%)	0 (0%)

^a^ Cases with more than one therapeutic indication for the same suspected drug.

### 3.1 Fall and fractures events

A total of 2,542 events were identified among ICSRs (N = 507), with a mean of 5.0 events per ICSR. All events are showed in Supplementary Table S2. Among these events, 653 were related to a fall or bone fracture (1.3 events per ICSR). In line with the trend of ICSRs, the majority of events was reported with canagliflozin (N = 237; 36.1%), followed by empagliflozin (N = 235; 35.8%), and dapagliflozin (N = 181; 27.5%). Fall was the most reported event with SGLT2 inhibitors (N = 333; 51.0%). A total of 189 out of 333 falls (56.84%) happened in presence of concomitant predisposing risk factors, such as treatment with cardiovascular medicines (*n* = 116), vertigo (n = 47), hypotension (*n* = 40), hypoglycemia (*n* = 16), atrial fibrillation (*n* = 5), and diabetic coma (*n* = 4). Cardiovascular medicines identified were beta-blockers, angiotensin receptor blockers, ACE inhibitors, antiplatelet, anticoagulants, diuretics, and alfa1-blockers. Overall, events of bone fractures with SGLT2 inhibitors were 320 (49.0%), with foot fractures (N = 40; 6.1%) and hip fractures (N = 32; 4.9%) as the most reported. Fall or factures events reported with each SGLT2 inhibitor are listed in Table 2. Most events were serious (N = 587; 89.9%) and classified as “caused or prolonged hospitalization” (N = 311; 47.6%) and “other medically important condition” (N = 219; 33.5%). The outcome of events was mostly unknown (N = 364; 55.7%). The 20.5% (N = 134) of events had a complete resolution and only the 2.9% (N = 19) was fatal. Seriousness and outcome criteria for each SGLT2 inhibitor were reported in Table 3. Fall and Fracture events reported with DPP4 inhibitors are shown in Supplementary Table S3. The information on TTO was available in 46 ICSRs with a median of 64 days (IQR: 127-21) for fall and 122 days (IQR: 384-77) for fractures.

**TABLE 2:** Fall and bone fractures listed in Individual Case Safety Reports (ICSRs) with SGLT2 inhibitors as suspected drugs and reported in Eudravigilance from January 1st. 2015 to December 31st. 2022.

	Canagliflozin (*N* = 237)	Empagliflozin (*N* = 235)	Dapagliflozin (*N* = 181)	Overall (*N* = 653)
Preferred terms				
Fall	100 (42.2%)	132 (56.2%)	101 (55.8%)	333 (51.0%)
Foot fracture	27 (11.4%)	5 (2.1%)	8 (4.4%)	40 (6.1%)
Hip fracture	10 (4.2%)	18 (7.7%)	4 (2.2%)	32 (4.9%)
Femur fracture	8 (3.4%)	9 (3.8%)	10 (5.5%)	27 (4.3%)
Ankle fracture	11 (4.6%)	7 (3.0%)	8 (4.4%)	26 (4.1%)
Fracture	14 (5.9%)	4 (1.7%)	6 (3.3%)	24 (3.7%)
Lower limb fracture	6 (2.5%)	8 (3.4%)	9 (5.0%)	23 (3.5%)
Upper limb fracture	10 (4.2%)	6 (2.6%)	7 (3.9%)	23 (3.5%)
Rib fracture	6 (2.5%)	6 (2.6%)	5 (2.8%)	17 (2.6%)
Humerus fracture	6 (2.5%)	4 (1.7%)	3 (1.7%)	13 (2.0%)
Spinal compression fracture	6 (2.5%)	3 (1.3%)	4 (2.2%)	13 (2.0%)
Wrist fracture	4 (1.7%)	2 (0.9%)	3 (1.7%)	9 (1.4%)
Femoral neck fracture	2 (0.8%)	4 (1.7%)	2 (1.1%)	8 (1.2%)
Tibia fracture	6 (2.5%)	1 (0.4%)	1 (0.6%)	8 (1.2%)
Compression fracture	3 (1.3%)	0 (0%)	2 (1.1%)	5 (0.8%)
Tooth fracture	0 (0%)	3 (1.3%)	2 (1.1%)	5 (0.8%)
Multiple fractures	0 (0%)	2 (0.9%)	2 (1.1%)	4 (0.6%)
Clavicle fracture	1 (0.4%)	2 (0.9%)	0 (0%)	3 (0.5%)
Fibula fracture	2 (0.8%)	1 (0.4%)	0 (0%)	3 (0.5%)
Forearm fracture	1 (0.4%)	2 (0.9%)	0 (0%)	3 (0.5%)
Hand fracture	1 (0.4%)	1 (0.4%)	1 (0.6%)	3 (0.5%)
Patella fracture	3 (1.3%)	0 (0%)	0 (0%)	3 (0.5%)
Radius fracture	1 (0.4%)	2 (0.9%)	0 (0%)	3 (0.5%)
Spinal fracture	2 (0.8%)	1 (0.4%)	0 (0%)	3 (0.5%)
Lumbar vertebral fracture	0 (0%)	3 (1.3%)	0 (0%)	3 (0.5%)
Fracture nonunion	2 (0.8%)	0 (0%)	0 (0%)	2 (0.3%)
Scapula fracture	1 (0.4%)	1 (0.4%)	0 (0%)	2 (0.3%)
Facial bones fracture	0 (0%)	1 (0.4%)	1 (0.6%)	2 (0.3%)
Comminuted fracture	1 (0.4%)	0 (0%)	0 (0%)	1 (0.1%)
Fracture displacement	1 (0.4%)	0 (0%)	0 (0%)	1 (0.1%)
Lisfranc fracture	1 (0.4%)	0 (0%)	0 (0%)	1 (0.1%)
Open fracture	1 (0.4%)	0 (0%)	0 (0%)	1 (0.1%)
Skull fracture	0 (0%)	0 (0%)	1 (0.6%)	1 (0.1%)
Traumatic fracture	0 (0%)	0 (0%)	1 (0.6%)	1 (0.1%)
Cervical vertebral fracture	0 (0%)	1 (0.4%)	0 (0%)	1 (0.1%)
Fractured coccyx	0 (0%)	1 (0.4%)	0 (0%)	1 (0.1%)
Pelvic fracture	0 (0%)	1 (0.4%)	0 (0%)	1 (0.1%)
Skull fractured base	0 (0%)	1 (0.4%)	0 (0%)	1 (0.1%)
Sternal fracture	0 (0%)	1 (0.4%)	0 (0%)	1 (0.1%)
Thoracic vertebral fracture	0 (0%)	1 (0.4%)	0 (0%)	1 (0.1%)
Ulna fracture	0 (0%)	1 (0.4%)	0 (0%)	1 (0.1%)

**TABLE 3:** Distribution for seriousness and outcome criteria of falls and fractures reported with SGLT2 inhibitors as suspected drugs and reported in Eudravigilance from January 1st. 2015 to December 31st. 2022.

	Canagliflozin (*N* = 237)	Empagliflozin (*N* = 235)	Dapagliflozin (*N* = 181)	Overall (*N* = 653)
Seriousness				
*Not serious*	1 (0.4%)	41 (17.4%)	24 (13.3%)	66 (10.1%)
*Caused/Prolonged Hospitalisation*	118 (49.8%)	120 (51.1%)	73 (40.3%)	311 (47.6%)
*Disabling*	5 (2.1%)	2 (0.9%)	2 (1.1%)	9 (1.4%)
*Life Threatening*	7 (3.0%)	15 (6.4%)	7 (3.9%)	29 (4.4%)
*Other Medically Important Condition*	99 (41.8%)	49 (20.9%)	71 (39.2%)	219 (33.5%)
*Results in Death*	7 (3.0%)	8 (3.4%)	4 (2.2%)	19 (2.9%)
Outcome				
*Fatal*	7 (3.0%)	8 (3.4%)	4 (2.2%)	19 (2.9%)
*Not Recovered/Not Resolved*	12 (5.1%)	25 (10.6%)	16 (8.8%)	53 (8.1%)
*Recovered/Resolved*	46 (19.4%)	53 (22.6%)	35 (19.3%)	134 (20.5%)
*Recovered/Resolved With Sequelae*	4 (1.7%)	3 (1.3%)	2 (1.1%)	9 (1.4%)
*Recovering/Resolving*	33 (13.9%)	21 (8.9%)	20 (11.0%)	74 (11.3%)
*Unknown*	135 (57.0%)	125 (53.2%)	104 (57.5%)	364 (55.7%)

### 3.2 Reporting frequencies of fall and bone fractures

**FIGURE 2:**
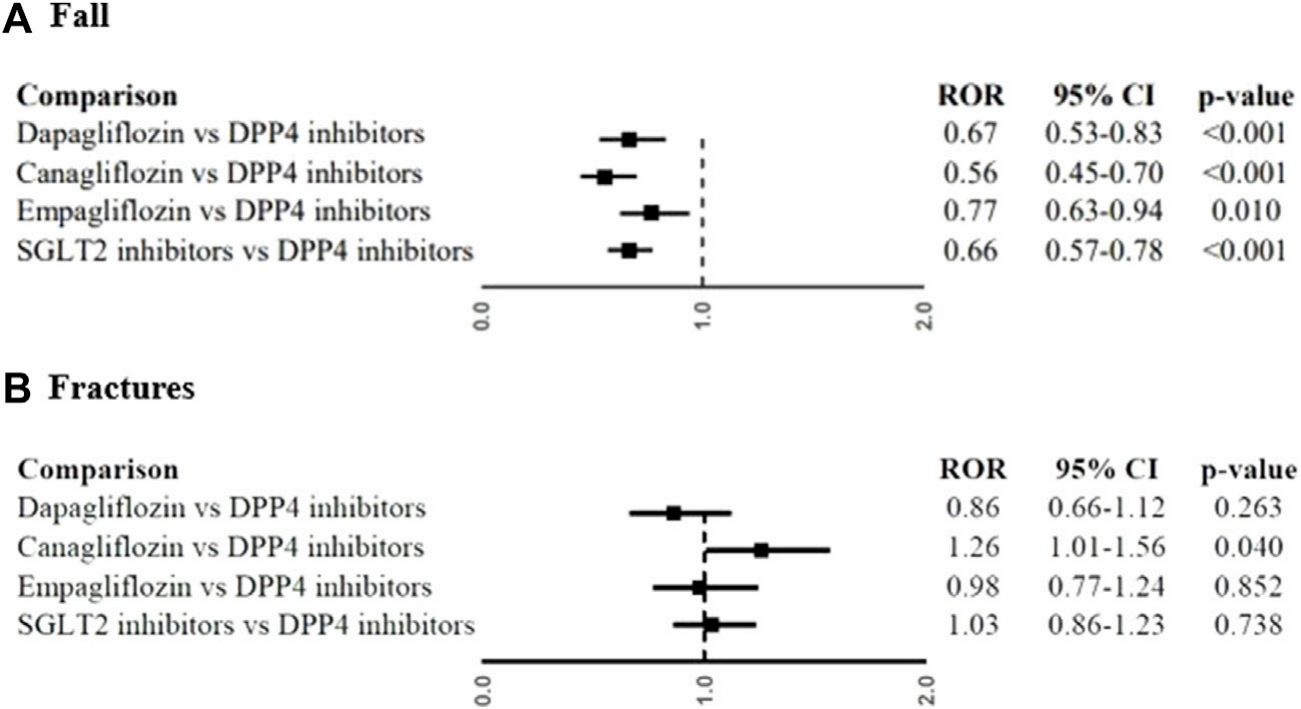
Reporting probabilities of fall **(A)** and fractures **(B)** between SGLT2 inhibitors and DPP4 inhibitors. Data were expressed as Reporting Odds Ratios (RORs) and their 95% Confidence Intervals (95%CI).

**FIGURE 3:**
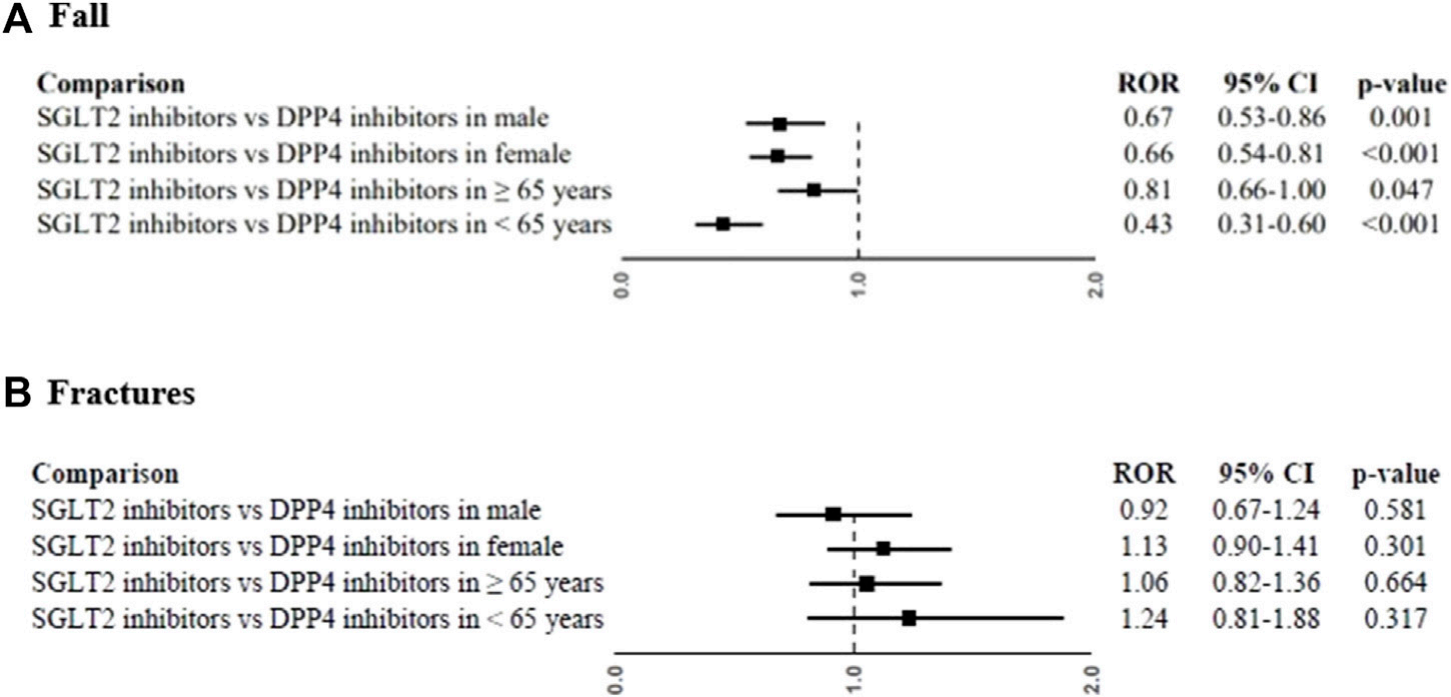
Reporting probabilities of fall **(A)** and fractures **(B)** between SGLT2 inhibitors and DPP4 inhibitors for sex (male and female) and age (≥65 years and <65 years). Data were expressed as Reporting Odds Ratios (RORs) and their 95% Confidence Intervals (95%CI).

SGLT2 inhibitors were associated with a lower reporting probability of fall than DPP4 inhibitors (ROR, 0.66; 95%CI, 0.57-0.78). The lower reporting probability of fall was also observed in the analysis with the single SGLT2 inhibitor: dapagliflozin (ROR, 0.67; 95%CI, 0.53-0.83), canagliflozin (ROR, 0.56; 95%CI, 0.45-0.70), and empagliflozin (ROR, 0.77; 95%CI, 0.63-0.94) compared to DPP4 inhibitors (Figure 2A). A lower reporting probability of fall was also found in the analyses for male (ROR, 0.67; 95%CI, 0.53-0.86; Figure 3A) and female (ROR, 0.66; 95%CI, 0.54-0.81; Figure 3A) when SGLT2 inhibitors were compared to DPP4 inhibitors. Moreover, the reporting probability of fall was lower with SGLT2 inhibitors than DPP4 inhibitors for cases with less than 65 years (ROR, 0.43; 95%CI, 0.31-0.60; Figure 3A), but not in those equal or greater than 65 years (ROR, 0.81; 95%CI, 0.66-1.00; Figure 3A). For fractures, canagliflozin compared with DPP4 inhibitors showed a slightly significant increased reporting (ROR, 1.26; 95%CI, 1.01-0.56; Figure 2B), but not confirmed in the sensitivity analysis (ROR, 1.14; 95%CI, 0.90-1.43; Supplementary Table S4). All other comparison showed no statistically significant difference for fracture reporting (Figure 2B). No difference for the reporting of fractures between SGLT2 inhibitors and DPP4 inhibitors was also found for gender and age group analyses (Figure 3B).

In the analyses within the class of SGLT2 inhibitors, female were associated with a higher reporting probability than male for both fall (ROR, 1.72; 95%CI, 1.38-2.14; *p*-value, <0.001) and fractures (ROR, 2.36; 95%CI, 1.86-2.98; *p*-value, <0.001). Moreover, the age ≥65 years was associated with a higher reporting probability of fall (ROR, 3.27; 95%CI, 2.54-4.20; *p*-value, <0.001) and fractures (ROR, 1.88; 95%CI, 1.47-2.40; *p*-value, <0.001) than <65 years.

The lower reporting probability of fall was found for cases without predisposing risk factors compared to those with predisposing risk factors for dapagliflozin (ROR, 0.27; 95%CI, 0.18-0.42), canagliflozin (ROR, 0.28; 95%CI, 0.19-0.42), and empagliflozin (ROR, 0.40; 95%CI, 0.28-0.56). All results of sensitivity analyses are shown in Supplementary Tables S4, S5.

## 4 Discussion

Despite limitation of our analyses performed on pool spontaneous reports of adverse events, in this real-world safety pharmacovigilance study, we found a 34% lower reporting probability of fall with SGLT-2 inhibitors compared with DPP4 inhibitors. Specifically, a 44% lower reporting probability of fall was found for canagliflozin, followed by dapagliflozin (33%), and empagliflozin (23%) in spite of the different year of marketing authorization. Indeed, dapagliflozin was the first authorized SGLT-2 inhibitor (Year 2012) in Europe, followed by canagliflozin (Year 2013) and empagliflozin (Year 2014). Although European consumption data on SGLT2 inhibitors are lacking, canagliflozin seems the most prescribed SGLT-2 inhibitor followed by empagliflozin and dapagliflozin (Sangha et al., 2021).

In Europe, falls are related to at least 3.8 million/year of visits in the emergency departments for fall-related injuries in older adults, of which 1.4 million generally require hospitalization (Promotion EAfIPaS, 2022). Falls negatively affect functionality and quality of life especially in elderly patients (Coles et al., 2020). Indeed, we found a higher reporting probability of falls for the age group ≥65 years than <65 years in SGLT2 inhibitors cases. It may also be related to the high prevalence of diabetes mellitus in older adults. It was estimated that 30% of people aged more than 65 years is affected by diabetes mellitus, with 90% of them having the type 2 (Custódio et al., 2020). However, when comparing the reporting probability of falls between SGLT-2 inhibitors and DPP4 inhibitors in cases aged ≥65 years, no difference was observed. This could be related to the notoriously higher frailty of older patients affected by diabetes mellitus and other cardiovascular comorbidities (Ungar et al., 2021; Wiersinga et al., 2021). Generally, patients treated with SGLT-2 inhibitors or DPP4 inhibitors are vulnerable and characterized by many predisposing risk factors for falls besides the anti-diabetic medication, including the presence of cardiovascular comorbidities that often require treatments with hypotensive agents. Hypotension is associated with a high risk of falls (Jansen et al., 2016) due to the acute transient hypoperfusion in some cerebral area that can cause neurodegenerative lesions and gait/balance problems (van Poelgeest et al., 2023). Hypotension due to volume depletion is an uncommon (1/1.000 ≤ frequency <1/100) event with SGLT2 inhibitors (European Medicines Agency EMA, 2023a; European Medicines Agency EMA, 2023b) and seems more frequent in patients co-treated with diuretics (van Poelgeest et al., 2023). Moreover, the increased diuresis than can occur with both medicines can evolve to urinary incontinence (Strain et al., 2021), which is another independent risk factor for falls (Deandrea et al., 2010) that can negatively impact quality of life in elders (Wehling, 2013; Benetos et al., 2019). We found that about half of falls (N = 116/336; 52.68%) happened in patients treated with anti-hypertensive drugs and that the association with falls was lower in the absence of risk factors. Based on the potential to cause or worsen the risk of fall, the prescription of SGLT2 inhibitors with diuretics or other anti-hypertensive drugs should be considered with caution at the lowest dosage possible, especially in elderly patients, and volume status, blood pressure, and electrolytes should be monitored during follow-up visits (van Poelgeest et al., 2023). Concerns were also raised for the potential ability of SGLT2 inhibitors to harm bone metabolism through the modulation of calcium and phosphate homeostasis (Blau and Taylor, 2018). Bone fragility can cause fractures and then falls. On the contrary, DPP4 inhibitors were hypothesized to have protective effects on bone by promoting osteoblast differentiation and inhibiting osteoclast activity (Kalaitzoglou et al., 2019; Hygum et al., 2020).

Although there are biologically plausible mechanisms underlying the onset of these adverse events with SGLT-2 inhibitors, we found a lower reporting probability of falls and no effect on the reporting probability of fractures (except for the slight increase with canagliflozin not confirmed in the sensitivity analysis) when these drugs were compared with DPP4 inhibitors. Clinical studies on this outcomes are inconsistent. The CANVAS trial found a higher rate of fractures (26%) with canagliflozin than placebo (Neal et al., 2015) (Hazard Ratio, HR, 1.26; 95%CI, 1.04-1.52). On the contrary, the Canagliflozin and Renal Outcomes in Type 2 Diabetes and Nephropathy trial and other large RCTs, as well as meta-analyses, did not find an increased risk of fractures (B et al., 2015; Ruanpeng et al., 2017; McMurray et al., 2019; Perkovic et al., 2019; Wiviott et al., 2019; Heerspink et al., 2020; Lou et al., 2020). Moreover, in accordance with our results, observational studies did not show an increased risk of fractures with SGLT2 inhibitors compared with DPP4 inhibitors (HR, 1.11; 95%CI, 0.96-1.28) (Adimadhyam et al., 2019) or GLP-1 receptor agonists (HR, 0.98; 95%CI, 0.75-1.26; HR, 1.11; 95%CI, 0.93-1.33) in relatively young (mean: 55–61 years) (Ueda et al., 2018; Fralick et al., 2019) or older patients (compared with DPP4 inhibitors: HR, 0.90; 95% CI, 0.73-1.11; or compared with GLP-1 receptor agonists: HR, 1.00; 95%CI, 0.80-1.25) (Zhuo et al., 2021). Finally, a US pharmacovigilance study found no signal for the reporting of fractures with canagliflozin (ROR: 0.49; 95%CI 0.42-0.57), dapagliflozin (ROR, 0.54; 95%CI, 0.43–0.67), and empagliflozin (ROR, 0.42; 95%CI 0.33-0.52) (Zhao et al., 2021). For the risk of fall, a pharmacovigilance studies conducted on the US FAERS found no difference in the reporting when SGLT-2 inhibitors were compered to other non-insulin antidiabetic drugs (ROR in adults, 1.16; 95%CI, 0.98-1.37 and ROR in older adults, 1.19; 95%CI, 0.89-1.59) (Goldman et al., 2023). According to our findings, instead, an observational study found a lower risk of falls with SGLT-2 inhibitors than DPP4 inhibitors (HR, 0.82; 95%CI, 0.77-0.87) (Zhuo et al., 2021). However, as also stated by study’s Authors, DPP4 inhibitors users were older and frailer than SGLT-2 inhibitors users before matching, thus suggesting that even after adjustment for many measured factors (such as frailty status and age) residual confounding factors may be present such as the presence of mild cognitive impairment (Zhuo et al., 2021). Therefore, in our study, we cannot exclude a higher frailty for cases with DPP4 inhibitors since the 67% of falls occurred in presence of other predisposing factors compared to the 53% with SGLT-2 inhibitors. Moreover, the adjustment in concomitant medications that can occur at the beginning of treatment with SGLT-2 inhibitors (such as deprescribing diuretics or insulin) (Li et al., 2019; van Poelgeest et al., 2023) might also justify the decreased risk of falls. Further studies may specifically clarify whether the initiation of SGLT-2 inhibitors is associated with deprescribing. Furthermore, falls and fractures are typically caused by the interaction of multiple confounders, such comorbidities (musculoskeletal, cardiovascular, metabolic, degenerative disorders, etc.) and drug therapies (cardiovascular, psychotropic medications, etc.) (Berry and Miller, 2008), which are difficult to measure with spontaneous reporting data.

In the analysis for gender, we found a higher reporting probability with female than male in accordance with the CANVAS and an observational study that found a high rate of fractures in female but not associated with a statistically significant increased risk (Watts et al., 2016; Zhuo et al., 2021). Furthermore, we need to consider in spite of the higher initiation of SGLT-2 inhibitors in male that female patients are more prone to develop adverse events with SGLT-2 inhibitors (Di Mauro et al., 2022), but also in general independently from the drug class due to hormone-related pharmacokinetics changes.

## 5 Strengths and limitations

The main strength is the use of the large EV database that allows to detect a wide range of real-life safety cases related to the treatment with SGLT2 inhibitors. The use of pharmacovigilance data is indeed not expensive and helpful to better characterize the drug safety profile. However, several limits need to be identified for the analysis of EV data. Firstly, safety reports may not be reported to national competent authorities and therefore in EV (underreporting), thus implying that not all safety cases that occurred in real-life are contained in EV. There are several reasons for failing to recognize an adverse events as linked to a drug. The common reasons for HCPs are the feeling of guilt, ignorance, lethargy, inadequate risk perception, diffidence, insufficient training, and lack of awareness about pharmacovigilance (Li et al., 2022). Another limitation is the quality of information reported that can be non-homogeneous and incomplete. Many cases may lack of information on age, drug dosing, time to onset, comorbid conditions, therapeutic indication, and concomitant drugs, thus hindering the identification of risk factors. Indeed, our descriptive analysis for risk factors may underestimate the real frailty among cases since many comorbidities or drug therapies may not be reported due to lack of time or laziness in completing the report form. This is a vary important limitation considering that falls and fractures can be caused by the interaction of multiple confounders. Moreover, another limitation is the impossibility to obtain data regarding the entirety of ICSRs reported in the EV database, thus impeding a proper evaluation of safety signals. Indeed, in contrast to other pharmacovigilance databases, we cannot retrieve all ICSRs on falls and bone fractures from the online EV (www.adrreports.eu), making impossible the computation of disproportionality analyses on all ICSRs. Furthermore, the exact denominator of patients exposed to SGLT2 inhibitors cannot be evaluated in EV, not allowing any estimate in terms of incidence. The total number of events for a drug can be used instead as a denominator for disproportionality analyses. Finally, in the absence of consumption and prescription data, we cannot exclude that differences in the reporting were related to a differential use.

## 6 Conclusion

In this European safety study, SGLT-2 inhibitors were associated with a lower reporting probability of falls compared with DPP4 inhibitors. Results were consistent across sex and age categories. Our results provide real-world evidence on the safety profile of SGLT-2 inhibitors. Generally, prior to initiate any treatment, clinicians must consider predisposing risk factors for potential adverse events before developing or adjusting a treatment pharmacological plan that includes SGLT2 inhibitors. Future researches and post-marketing analyses involving these drugs will help to understand their long-term safety.

## Data Availability

Publicly available datasets were analyzed in this study. This data can be found here: https://www.adrreports.eu/.
