# Supplementary material for: Sodium-glucose cotransporter–2 (SGLT2) inhibitors and the reporting of falls and fractures: an european pharmacovigilance analysis
Source: Front Pharmacol. 2023 Nov 6;14:1245642. doi: 10.3389/fphar.2023.1245642 (PMC10657831; doi:10.3389/fphar.2023.1245642)
Supplement: Supplementary file 1 [file DataSheet1.docx]

**Table S1.** List of preferred terms (PTs) searched for the analysis.

| **Preferred terms** |
| --- |
| Fall |
| Acetabulum fracture |
| Alveolar border of body of mandible closed fracture |
| Alveolar border of body of mandible open fracture |
| Angle of jaw closed fracture |
| Angle of jaw open fracture |
| Ankle fracture |
| Barton's fracture |
| Bennet's fracture |
| Bimalleolar fracture, closed |
| Bimalleolar fracture, open |
| Blow out fracture of orbit |
| Body of mandible of other and unspecified part, open fracture |
| Bone fracture (not spontaneous) |
| Boxer's fracture |
| Burst fracture |
| Clavicle fracture |
| Closed fracture of acetabulum |
| Closed fracture of acromial end of clavicle |
| Closed fracture of acromial process of scapula |
| Closed fracture of base of other metacarpal bone(s) |
| Closed fracture of base of skull with cerebral laceration and contusion |
| Closed fracture of base of skull with intracranial injury |
| Closed fracture of base of skull with intracranial injury of other and unspecified nature |
| Closed fracture of base of skull with other and unspecified intracranial hemorrhage |
| Closed fracture of base of skull with subarachnoid, subdural, and extradural hemorrhage |
| Closed fracture of base of skull without mention of intracranial injury |
| Closed fracture of base of thumb (first) metacarpal |
| Closed fracture of c1-c4 level with anterior cord syndrome |
| Closed fracture of c1-c4 level with central cord syndrome |
| Closed fracture of c1-c4 level with complete lesion of cord |
| Closed fracture of c1-c4 level with other specified spinal cord injury |
| Closed fracture of c1-c4 level with unspecified spinal cord injury |
| Closed fracture of c5-c7 level with anterior cord syndrome |
| Closed fracture of c5-c7 level with central cord syndrome |
| Closed fracture of c5-c7 level with complete lesion of cord |
| Closed fracture of c5-c7 level with other specified spinal cord injury |
| Closed fracture of c5-c7 level with unspecified spinal cord injury |
| Closed fracture of capitate bone (os magnum) of wrist |
| Closed fracture of carpal bone, unspecified |
| Closed fracture of cervical vertebra with spinal cord injury |
| Closed fracture of cervical vertebra with spinal cord lesion |
| Closed fracture of cervical vertebra without mention of spinal cord injury |
| Closed fracture of cervical vertebra without mention of spinal cord lesion |
| Closed fracture of cervical vertebra, unspecified level |
| Closed fracture of clavicle |
| Closed fracture of clavicle, unspecified part |
| Closed fracture of coracoid process of scapula |
| Closed fracture of distal phalanx or phalanges of hand |
| Closed fracture of dorsal (thoracic) vertebra without mention of spinal cord injury |
| Closed fracture of dorsal (thoracic) vertebra without mention of spinal cord lesion |
| Closed fracture of dorsal vertebra with spinal cord injury |
| Closed fracture of dorsal vertebra with spinal cord lesion |
| Closed fracture of eight or more ribs |
| Closed fracture of fifth cervical vertebra |
| Closed fracture of first cervical vertebra |
| Closed fracture of five ribs |
| Closed fracture of four ribs |
| Closed fracture of fourth cervical vertebra |
| Closed fracture of glenoid cavity and neck of scapula |
| Closed fracture of hamate (unciform) bone of wrist |
| Closed fracture of ilium |
| Closed fracture of ischium |
| Closed fracture of larynx and trachea |
| Closed fracture of lumbar spine with spinal cord injury |
| Closed fracture of lumbar spine with spinal cord lesion |
| Closed fracture of lumbar vertebra without mention of spinal cord injury |
| Closed fracture of lumbar vertebra without mention of spinal cord lesion |
| Closed fracture of lunate (semilunar) bone of wrist |
| Closed fracture of metacarpal bone(s), site unspecified |
| Closed fracture of metacarpal bones |
| Closed fracture of middle or proximal phalanx or phalanges of hand |
| Closed fracture of multiple cervical vertebrae |
| Closed fracture of multiple ribs, unspecified |
| Closed fracture of multiple sites of metacarpus |
| Closed fracture of multiple sites of phalanx or phalanges of hand |
| Closed fracture of navicular (scaphoid) bone of wrist |
| Closed fracture of neck of metacarpal bone(s) |
| Closed fracture of one or more phalanges of foot |
| Closed fracture of one or more phalanges of hand |
| Closed fracture of one rib |
| Closed fracture of other bone of wrist |
| Closed fracture of other part of scapula |
| Closed fracture of patella |
| Closed fracture of phalanx or phalanges of hand, unspecified |
| Closed fracture of pisiform bone of wrist |
| Closed fracture of pubis |
| Closed fracture of rib(s) |
| Closed fracture of rib(s), unspecified |
| Closed fracture of sacrum and coccyx with complete cauda equina lesion |
| Closed fracture of sacrum and coccyx with other cauda equina injury |
| Closed fracture of sacrum and coccyx with other spinal cord injury |
| Closed fracture of sacrum and coccyx with spinal cord injury |
| Closed fracture of sacrum and coccyx with spinal cord lesion |
| Closed fracture of sacrum and coccyx with unspecified spinal cord injury |
| Closed fracture of sacrum and coccyx without mention of spinal cord injury |
| Closed fracture of sacrum and coccyx without mention of spinal cord lesion |
| Closed fracture of scapula |
| Closed fracture of scapula, unspecified part |
| Closed fracture of second cervical vertebra |
| Closed fracture of seven ribs |
| Closed fracture of seventh cervical vertebra |
| Closed fracture of shaft of clavicle |
| Closed fracture of shaft of fibula |
| Closed fracture of shaft of fibula with tibia |
| Closed fracture of shaft of metacarpal bone(s) |
| Closed fracture of shaft of tibia |
| Closed fracture of shaft or unspecified part of humerus |
| Closed fracture of six ribs |
| Closed fracture of sixth cervical vertebra |
| Closed fracture of sternal end of clavicle |
| Closed fracture of sternum |
| Closed fracture of t1-t6 level with anterior cord syndrome |
| Closed fracture of t1-t6 level with central cord syndrome |
| Closed fracture of t1-t6 level with complete lesion of cord |
| Closed fracture of t1-t6 level with other specified spinal cord injury |
| Closed fracture of t1-t6 level with unspecified spinal cord injury |
| Closed fracture of t7-t12 level with anterior cord syndrome |
| Closed fracture of t7-t12 level with central cord syndrome |
| Closed fracture of t7-t12 level with complete lesion of cord |
| Closed fracture of t7-t12 level with other specified spinal cord injury |
| Closed fracture of t7-t12 level with unspecified spinal cord injury |
| Closed fracture of third cervical vertebra |
| Closed fracture of three ribs |
| Closed fracture of trapezium bone (larger multangular) of wrist |
| Closed fracture of trapezoid bone (smaller multangular) of wrist |
| Closed fracture of triquetral (cuneiform) bone of wrist |
| Closed fracture of two ribs |
| Closed fracture of unspecified part of fibula |
| Closed fracture of unspecified part of fibula with tibia |
| Closed fracture of unspecified part of forearm |
| Closed fracture of unspecified part of tibia |
| Closed fracture of unspecified part of vertebral column without mention of spinal cord injury |
| Closed fracture of unspecified part of vertebral column without mention of spinal cord lesion |
| Closed fracture of unspecified vertebra with spinal cord injury |
| Closed fracture of unspecified vertebra with spinal cord lesion |
| Closed fracture of upper end of fibula |
| Closed fracture of upper end of fibula with tibia |
| Closed fracture of upper end of tibia |
| Closed fracture of vault of skull with cerebral laceration and contusion |
| Closed fracture of vault of skull with intercranial injury of other and unspecified nature |
| Closed fracture of vault of skull with intracranial injury |
| Closed fracture of vault of skull with other and unspecified intracranial hemorrhage |
| Closed fracture of vault of skull with subarachnoid, subdural, and extradural hemorrhage |
| Closed fracture of vault of skull without mention of intracranial injury |
| Closed fractures involving skull or face with other bones, with cerebral laceration and contusion |
| Closed fractures involving skull or face with other bones, with intracranial injury |
| Closed fractures involving skull or face with other bones, without mention of intracranial injury |
| Closed fractures of carpal bones |
| Closed skull fracture with intracranial injury of other and unspecified nature |
| Closed skull fracture with other and unspecified intracranial hemorrhage |
| Closed, fracture of shaft of humerus |
| Closed, fracture of unspecified part of humerus |
| Colles' fracture |
| Colles' fracture, open |
| Complicated fracture |
| Compound fracture |
| Compound fracture - femur |
| Compound fracture - fibula |
| Compound fracture - humerus |
| Compound fracture - radius |
| Compound fracture - tibia |
| Compound fracture - ulna |
| Compression fracture |
| Compression of fractured vertebra |
| Condylar process of mandible open fracture |
| Condylar process of mandible, closed fracture |
| Coronoid process of mandible closed fracture |
| Coronoid process of mandible open fracture |
| Elbow fracture |
| Facial bones fracture |
| Femoral neck fracture |
| Femur fracture |
| Femur fracture (excl neck) |
| Femur fracture NOS |
| Fibula fracture |
| Foot fracture |
| Forearm fracture |
| Fracture |
| Fracture bone |
| Fracture closed of lower end of forearm, unspecified |
| Fracture closed of upper end of forearm, unspecified |
| Fracture Colles' |
| Fracture femur |
| Fracture Monteggia's, closed |
| Fracture multiple |
| Fracture NOS |
| Fracture of anatomical neck of humerus, closed |
| Fracture of anatomical neck of humerus, open |
| Fracture of ankle |
| Fracture of ankle, unspecified, closed |
| Fracture of astragalus, closed |
| Fracture of astragalus, open |
| Fracture of base of neck of femur, closed |
| Fracture of base of neck of femur, open |
| Fracture of base of skull |
| Fracture of base of skull, closed with intracranial injury |
| Fracture of base of skull, open with intracranial injury |
| Fracture of bones of trunk, closed |
| Fracture of bones of trunk, open |
| Fracture of calcaneus, closed |
| Fracture of calcaneus, open |
| Fracture of carpal bone(s) |
| Fracture of clavicle |
| Fracture of clavicle due to birth trauma |
| Fracture of clavicle, closed |
| Fracture of clavicle, open |
| Fracture of coronoid process of ulna, closed |
| Fracture of coronoid process of ulna, open |
| Fracture of cuboid bone, closed |
| Fracture of cuboid bone, open |
| Fracture of cuneiform bone of foot, closed |
| Fracture of cuneiform bone of foot, open |
| Fracture of distal end of ulna (alone), closed |
| Fracture of distal end of ulna (alone), open |
| Fracture of epiphysis (separation) (upper) of neck of femur, closed |
| Fracture of epiphysis (separation) (upper) of neck of femur, open |
| Fracture of face bones |
| Fracture of femoral condyle, closed |
| Fracture of femoral condyle, open |
| Fracture of greater tuberosity of humerus, closed |
| Fracture of greater tuberosity of humerus, open |
| Fracture of head of radius, closed |
| Fracture of head of radius, open |
| Fracture of humerus |
| Fracture of humerus, supracondylar closed |
| Fracture of humerus, supracondylar open |
| Fracture of intertrochanteric section of femur, closed |
| Fracture of intertrochanteric section of femur, open |
| Fracture of larynx and trachea, closed |
| Fracture of larynx and trachea, open |
| Fracture of lateral condyle of humerus, closed |
| Fracture of lateral condyle of humerus, open |
| Fracture of lateral malleolus, closed |
| Fracture of lateral malleolus, open |
| Fracture of lower end of femur, closed |
| Fracture of lower end of femur, open |
| Fracture of lower end of femur, unspecified part, closed |
| Fracture of lower end of femur, unspecified part, open |
| Fracture of lower end of humerus, closed |
| Fracture of lower end of humerus, open |
| Fracture of lower end of radius and ulna, closed |
| Fracture of lower end of radius and ulna, open |
| Fracture of lower end of radius with ulna, closed |
| Fracture of lower end of radius with ulna, open |
| Fracture of lower epiphysis of femur, closed |
| Fracture of lower epiphysis of femur, open |
| Fracture of medial condyle of humerus, closed |
| Fracture of medial condyle of humerus, open |
| Fracture of medial malleolus, closed |
| Fracture of medial malleolus, open |
| Fracture of metacarpal bone(s) |
| Fracture of metacarpal bones, closed |
| Fracture of metacarpal bones, open |
| Fracture of metatarsal bone(s), closed |
| Fracture of metatarsal bone(s), open |
| Fracture of midcervical section of femur, closed |
| Fracture of midcervical section of femur, open |
| Fracture of navicular (scaphoid) bone of foot, closed |
| Fracture of navicular (scaphoid) bone of foot, open |
| Fracture of neck of femur |
| Fracture of neck of radius, closed |
| Fracture of neck of radius, open |
| Fracture of olecranon process of ulna, closed |
| Fracture of olecranon process of ulna, open |
| Fracture of one or more phalanges of foot |
| Fracture of one or more phalanges of foot, closed |
| Fracture of one or more phalanges of foot, open |
| Fracture of one or more phalanges of hand |
| Fracture of one or more phalanges of hand, closed |
| Fracture of one or more phalanges of hand, open |
| Fracture of one or more tarsal and metatarsal bones |
| Fracture of other and unspecified parts of femur |
| Fracture of other specified part of pelvis, closed |
| Fracture of other specified part of pelvis, open |
| Fracture of other tarsal and metatarsal bones, closed |
| Fracture of other tarsal and metatarsal bones, open |
| Fracture of patella |
| Fracture of patella, closed |
| Fracture of patella, open |
| Fracture of pelvis |
| Fracture of pelvis, unspecified, closed |
| Fracture of pelvis, unspecified, open |
| Fracture of pubis, open |
| Fracture of radius and ulna |
| Fracture of radius with ulna, upper end (any part), closed |
| Fracture of radius with ulna, upper end (any part), open |
| Fracture of rib(s), open |
| Fracture of rib(s), sternum, larynx, and trachea |
| Fracture of scapula |
| Fracture of scapula, closed |
| Fracture of scapula, open |
| Fracture of shaft of femur, closed |
| Fracture of shaft of femur, open |
| Fracture of shaft of humerus, open |
| Fracture of shaft of radius (alone), closed |
| Fracture of shaft of radius (alone), open |
| Fracture of shaft of radius and ulna, closed |
| Fracture of shaft of radius and ulna, open |
| Fracture of shaft of radius or ulna, unspecified, closed |
| Fracture of shaft of radius or ulna, unspecified, open |
| Fracture of shaft of radius with ulna, closed |
| Fracture of shaft of radius with ulna, open |
| Fracture of shaft of tibia and fibula, closed |
| Fracture of shaft of tibia and fibula, open |
| Fracture of shaft of ulna (alone), closed |
| Fracture of shaft of ulna (alone), open |
| Fracture of shaft or unspecified part of femur, closed |
| Fracture of shaft or unspecified part of femur, open |
| Fracture of shaft or unspecified part of humerus, open |
| Fracture of spine |
| Fracture of sternum, closed |
| Fracture of sternum, open |
| Fracture of subtrochanteric section of femur, closed |
| Fracture of subtrochanteric section of femur, open |
| Fracture of surgical neck of humerus, closed |
| Fracture of surgical neck of humerus, open |
| Fracture of tibia and fibula |
| Fracture of unspecified bone(s) of foot (except toes), closed |
| Fracture of unspecified bone(s) of foot (except toes), open |
| Fracture of unspecified bone, closed |
| Fracture of unspecified bone, open |
| Fracture of unspecified bones |
| Fracture of unspecified condyle(s) of humerus, closed |
| Fracture of unspecified condyle(s) of humerus, open |
| Fracture of unspecified intracapsular section of neck of femur, closed |
| Fracture of unspecified intracapsular section of neck of femur, open |
| Fracture of unspecified part of femur, closed |
| Fracture of unspecified part of femur, open |
| Fracture of unspecified part of forearm, open |
| Fracture of unspecified part of humerus, open |
| Fracture of unspecified part of lower end of humerus, closed |
| Fracture of unspecified part of lower end of humerus, open |
| Fracture of unspecified part of neck of femur, closed |
| Fracture of unspecified part of neck of femur, open |
| Fracture of unspecified part of pelvis, closed |
| Fracture of unspecified part of pelvis, open |
| Fracture of unspecified part of radius (alone), closed |
| Fracture of unspecified part of radius (alone), open |
| Fracture of unspecified part of radius with ulna, closed |
| Fracture of unspecified part of radius with ulna, open |
| Fracture of unspecified part of tibia and fibula, closed |
| Fracture of unspecified part of tibia and fibula, open |
| Fracture of unspecified part of ulna (alone), closed |
| Fracture of unspecified part of ulna (alone), open |
| Fracture of unspecified part of upper end of humerus, closed |
| Fracture of unspecified part of upper end of humerus, open |
| Fracture of unspecified trochanteric section of femur, closed |
| Fracture of unspecified trochanteric section of femur, open |
| Fracture of upper end of humerus, closed |
| Fracture of upper end of humerus, open |
| Fracture of upper end of radius and ulna, closed |
| Fracture of upper end of radius and ulna, open |
| Fracture of upper end of tibia and fibula, closed |
| Fracture of upper end of tibia and fibula, open |
| Fracture of upper end or unspecified of tibia and fibula, open |
| Fracture of upper end or unspecified part of radius and ulna, closed |
| Fracture of upper end or unspecified part of radius and ulna, open |
| Fracture of upper end or unspecified part of tibia and fibula, closed |
| Fracture of vault of skull |
| Fracture of vault of skull, closed with intracranial injury |
| Fracture of vault of skull, open with intracranial injury |
| Fracture of vault of skull, open without intracranial injury |
| Fracture of vertebra |
| Fracture of vertebral column with spinal cord injury |
| Fracture of vertebral column with spinal cord lesion |
| Fracture of vertebral column without mention of spinal cord injury |
| Fracture of vertebral column without mention of spinal cord lesion |
| Fracture open of upper end of forearm, unspecified |
| Fracture rib |
| Fracture vertebral |
| Fracture, closed, colles' |
| Fracture, Monteggia's open |
| Fractured carpal |
| Fractured cervical spine |
| Fractured collar bone |
| Fractured compression T8 |
| Fractured ethmoid |
| Fractured femoral neck |
| Fractured femur (excl neck) |
| Fractured finger |
| Fractured hip |
| Fractured humerus (compound) |
| Fractured iliac crest |
| Fractured ischium |
| Fractured jaw |
| Fractured mandible |
| Fractured maxilla |
| Fractured metacarpal |
| Fractured metatarsal |
| Fractured neck of femur |
| Fractured nose |
| Fractured olecranon |
| Fractured os calcis |
| Fractured pelvis NOS |
| Fractured pisiform |
| Fractured radial head |
| Fractured ribs |
| Fractured sacrum |
| Fractured scaphoid |
| Fractured skull depressed |
| Fractured sternum |
| Fractured temporal bone |
| Fractured thumb |
| Fractured toe |
| Fractured transverse process |
| Fractured ulnar styloid process |
| Fractured vertebra |
| Fractured vertebra (compression) |
| Fractured wrist |
| Fractured zygoma |
| Fractures of proximal end of radius (alone), other and unspecified closed |
| Great toe fracture |
| Greenstick fracture |
| Hand fracture |
| Hip fracture |
| Humerus fracture |
| Ilium fracture |
| Ill-defined closed fractures of upper limb |
| Ill-defined fractures of bones of trunk |
| Ill-defined fractures of upper limb |
| Ill-defined fractures of upper limb, closed |
| Ill-defined fractures of upper limb, open |
| Ill-defined open fractures of upper limb |
| Jaw fracture |
| Jefferson's fracture |
| Late effect of fracture of lower extremities |
| Late effect of fracture of multiple and unspecified bones |
| Late effect of fracture of neck of femur |
| Late effect of fracture of skull and face bones |
| Late effect of fracture of spine and trunk without mention of spinal cord lesion |
| Late effect of fracture of upper extremities |
| Late effect of intracranial injury without mention of skull fracture |
| Leg fracture |
| Malar and maxillary bones, closed fracture |
| Malar and maxillary bones, open fracture |
| Mandible closed fracture |
| Mandible open fracture |
| March fracture |
| Multiple closed fractures involving both upper limbs, and upper limb with rib(s) and sternum |
| Multiple closed fractures of hand bones |
| Multiple closed pelvic fractures with disruption of pelvic circle |
| Multiple fractures |
| Multiple fractures involving both lwr limbs, lwr with upr limb, and lwr limb with rib and sternum |
| Multiple fractures involving both upper limbs, and upper limb with rib(s) and sternum |
| Multiple fractures involving both upper limbs, and upper limb with ribs and sternum |
| Multiple fractures involving skull or face with other bones |
| Multiple fractures of hand bones |
| Multiple fractures of hand bones, closed |
| Multiple fractures of hand bones, open |
| Multiple injuries (excl fractures) |
| Multiple open fractures involving both upper limbs, and upper limb with rib(s) and sternum |
| Multiple open fractures of hand bones |
| Multiple open pelvic fractures with disruption of pelvic circle |
| Multiple sites of mandible closed fracture |
| Multiple sites of mandible open fracture |
| Nasal bones, closed fracture |
| Nasal bones, open fracture |
| Navicular fracture |
| Olecranon fracture |
| Open fracture |
| Open fracture of acetabulum |
| Open fracture of acromial end of clavicle |
| Open fracture of acromial process of scapula |
| Open fracture of base of other metacarpal bone(s) |
| Open fracture of base of skull with cerebral laceration and contusion |
| Open fracture of base of skull with cerebral laceration and contusion, with concussion, unspecified |
| Open fracture of base of skull with cerebral laceration and contusion, with no loss of consciousness |
| Open fracture of base of skull with intracranial injury |
| Open fracture of base of skull with intracranial injury of other and unspecified nature |
| Open fracture of base of skull with other and unspecified intracranial hemorrhage |
| Open fracture of base of skull with subarachnoid, subdural, and extradural hemorrhage |
| Open fracture of base of skull without mention of intracranial injury |
| Open fracture of base of skull without mention of intracranial injury, with concussion, unspecified |
| Open fracture of base of skull without mention of intracranial injury, with no loss of consciousness |
| Open fracture of base of thumb (first) metacarpal |
| Open fracture of c1-c4 level with anterior cord syndrome |
| Open fracture of c1-c4 level with central cord syndrome |
| Open fracture of c1-c4 level with complete lesion of cord |
| Open fracture of c1-c4 level with other specified spinal cord injury |
| Open fracture of c1-c4 level with unspecified spinal cord injury |
| Open fracture of c5-c7 level with anterior cord syndrome |
| Open fracture of c5-c7 level with central cord syndrome |
| Open fracture of c5-c7 level with complete lesion of cord |
| Open fracture of c5-c7 level with other specified spinal cord injury |
| Open fracture of c5-c7 level with unspecified spinal cord |
| Open fracture of C5-C7 level with unspecified spinal cord injury |
| Open fracture of capitate bone (os magnum) of wrist |
| Open fracture of carpal bone, unspecified |
| Open fracture of cervical vertebra with spinal cord injury |
| Open fracture of cervical vertebra with spinal cord lesion |
| Open fracture of cervical vertebra without mention of spinal cord injury |
| Open fracture of cervical vertebra without mention of spinal cord lesion |
| Open fracture of cervical vertebra, unspecified level |
| Open fracture of clavicle |
| Open fracture of clavicle, unspecified part |
| Open fracture of coracoid process |
| Open fracture of distal phalanx or phalanges of hand |
| Open fracture of dorsal (thoracic) vertebra without mention of spinal cord injury |
| Open fracture of dorsal (thoracic) vertebra without mention of spinal cord lesion |
| Open fracture of dorsal vertebra with spinal cord injury |
| Open fracture of dorsal vertebra with spinal cord lesion |
| Open fracture of eight or more ribs |
| Open fracture of fifth cervical vertebra |
| Open fracture of first cervical vertebra |
| Open fracture of five ribs |
| Open fracture of four ribs |
| Open fracture of fourth cervical vertebra |
| Open fracture of glenoid cavity and neck of scapula |
| Open fracture of hamate (unciform) bone of wrist |
| Open fracture of ilium |
| Open fracture of ischium |
| Open fracture of larynx and trachea |
| Open fracture of lower end of forearm, unspecified |
| Open fracture of lumbar spine with spinal cord injury |
| Open fracture of lumbar spine with spinal cord lesion |
| Open fracture of lumbar vertebra without mention of spinal cord injury |
| Open fracture of lumbar vertebra without mention of spinal cord lesion |
| Open fracture of lunate (semilunar) bone of wrist |
| Open fracture of metacarpal bone(s), site unspecified |
| Open fracture of metacarpal bones |
| Open fracture of middle or proximal phalanx or phalanges of hand |
| Open fracture of multiple cervical vertebrae |
| Open fracture of multiple ribs, unspecified |
| Open fracture of multiple sites of metacarpus |
| Open fracture of multiple sites of phalanx or phalanges of hand |
| Open fracture of navicular (scaphoid) bone of wrist |
| Open fracture of neck of metacarpal bone(s) |
| Open fracture of one or more phalanges of foot |
| Open fracture of one or more phalanges of hand |
| Open fracture of one rib |
| Open fracture of other bone of wrist |
| Open fracture of other part of scapula |
| Open fracture of other tarsal and metatarsal bones |
| Open fracture of patella |
| Open fracture of phalanx or phalanges of hand, unspecified |
| Open fracture of pisiform bone of wrist |
| Open fracture of pubis |
| Open fracture of rib(s) |
| Open fracture of rib(s), unspecified |
| Open fracture of sacrum and coccyx with complete cauda equina lesion |
| Open fracture of sacrum and coccyx with other cauda equina injury |
| Open fracture of sacrum and coccyx with other spinal cord injury |
| Open fracture of sacrum and coccyx with spinal cord injury |
| Open fracture of sacrum and coccyx with spinal cord lesion |
| Open fracture of sacrum and coccyx with unspecified spinal cord injury |
| Open fracture of sacrum and coccyx without mention of spinal cord injury |
| Open fracture of sacrum and coccyx without mention of spinal cord lesion |
| Open fracture of scapula |
| Open fracture of scapula, unspecified part |
| Open fracture of second cervical vertebra |
| Open fracture of seven ribs |
| Open fracture of seventh cervical vertebra |
| Open fracture of shaft of clavicle |
| Open fracture of shaft of fibula |
| Open fracture of shaft of fibula with tibia |
| Open fracture of shaft of metacarpal bone(s) |
| Open fracture of shaft of tibia |
| Open fracture of six ribs |
| Open fracture of sixth cervical vertebra |
| Open fracture of sternal end of clavicle |
| Open fracture of sternum |
| Open fracture of t1-t6 level with anterior cord syndrome |
| Open fracture of t1-t6 level with central cord syndrome |
| Open fracture of t1-t6 level with complete lesion of cord |
| Open fracture of t1-t6 level with other specified spinal cord injury |
| Open fracture of t1-t6 level with unspecified spinal cord injury |
| Open fracture of t7-t12 level with anterior cord syndrome |
| Open fracture of t7-t12 level with central cord syndrome |
| Open fracture of t7-t12 level with complete lesion of cord |
| Open fracture of t7-t12 level with other specified spinal cord injury |
| Open fracture of t7-t12 level with unspecified spinal cord injury |
| Open fracture of third cervical vertebra |
| Open fracture of three ribs |
| Open fracture of trapezium bone (larger multangular) of wrist |
| Open fracture of trapezoid bone (smaller multangular) of wrist |
| Open fracture of triquetral (cuneiform) bone of wrist |
| Open fracture of two ribs |
| Open fracture of unspecified part of fibula |
| Open fracture of unspecified part of fibula with tibia |
| Open fracture of unspecified part of tibia |
| Open fracture of unspecified part of vertebral column without mention of spinal cord injury |
| Open fracture of unspecified part of vertebral column without mention of spinal cord lesion |
| Open fracture of unspecified vertebra with spinal cord injury |
| Open fracture of unspecified vertebra with spinal cord lesion |
| Open fracture of upper end of fibula |
| Open fracture of upper end of fibula with tibia |
| Open fracture of upper end of tibia |
| Open fracture of vault of skull with cerebral laceration and contusion |
| Open fracture of vault of skull with cerebral laceration and contusion, with concussion, unspecified |
| Open fracture of vault of skull with intracranial injury |
| Open fracture of vault of skull with intracranial injury of other and unspecified nature |
| Open fracture of vault of skull with other and unspecified intracranial hemorrhage |
| Open fracture of vault of skull with subarachnoid, subdural, and extradural hemorrhage |
| Open fracture of vault of skull with subarachnoid, subdural, and extradural hemorrhage, with |
| Open fracture of vault of skull without intracranial injury |
| Open fracture of vault of skull without mention of intra-cranial injury |
| Open fracture of vault of skull without mention of intracranial injury, with concussion, unspecified |
| Open fractures involving skull or face with other bones, with cerebral laceration and contusion |
| Open fractures involving skull or face with other bones, with intracranial injury |
| Open fractures involving skull or face with other bones, without mention of intracranial injury |
| Open fractures of carpal bones |
| Orbital floor (blow-out) closed fracture |
| Orbital floor (blow-out) open fracture |
| Other and unqualified skull fractures |
| Other and unspecified closed fractures of proximal end of ulna (alone) |
| Other and unspecified open fractures of proximal end of radius (alone) |
| Other and unspecified open fractures of proximal end of ulna (alone) |
| Other closed fractures of distal end of radius (alone) |
| Other closed fractures of lower end of humerus |
| Other closed fractures of upper end of humerus |
| Other closed skull fracture with cerebral laceration and contusion |
| Other closed skull fracture with cerebral laceration and contusion, with concussion, unspecified |
| Other closed skull fracture with cerebral laceration and contusion, with no loss of consciousness |
| Other closed skull fracture with intracranial injury |
| Other closed skull fracture with intracranial injury of other and unspecified nature |
| Other closed skull fracture with subarachnoid, subdural, and extradural hemorrhage |
| Other closed skull fracture without mention of intracranial injury |
| Other closed skull fracture without mention of intracranial injury, with concussion, unspecified |
| Other closed skull fracture without mention of intracranial injury, with no loss of consciousness |
| Other facial bones closed fracture |
| Other facial bones open fracture |
| Other fracture of lower end of femur, closed |
| Other fracture of lower end of femur, open |
| Other fracture of lower end of humerus, open |
| Other fracture of tarsal and metatarsal bones, closed |
| Other fractures of tarsal and metatarsal bones, open |
| Other ill-defined fractures of lower limb |
| Other open fracture of upper end of humerus |
| Other open fractures of distal end of radius (alone) |
| Other open skull fracture with cerebral laceration and contusion |
| Other open skull fracture with cerebral laceration and contusion, with concussion, unspecified |
| Other open skull fracture with cerebral laceration and contusion, with no loss of consciousness |
| Other open skull fracture with intracranial injury |
| Other open skull fracture with intracranial injury of other and unspecified nature |
| Other open skull fracture with other and unspecified intra-cranial hemorrhage |
| Other open skull fracture with subarachnoid, subdural, and extradural hemorrhage |
| Other open skull fracture without mention of injury, with state of consciousness unspecified |
| Other open skull fracture without mention of intracranial Injury |
| Other open skull fracture without mention of intracranial injury, with concussion, unspecified |
| Other open skull fracture without mention of intracranial injury, with no loss of consciousness |
| Other transcervical fracture of femur, closed |
| Other transcervical fracture of femur, open |
| Other, multiple and ill-defined fractures of lower limb, closed |
| Other, multiple and ill-defined fractures of lower limb, open |
| Other, multiple, and ill-defined fractures of lower limb |
| Part of body of mandible closed fracture of other and unspecified part |
| Patella fracture |
| Pertrochanteric fracture of femur, closed |
| Pertrochanteric fracture of femur, open |
| Pott's fracture |
| Pubic rami fracture |
| Radial head fracture |
| Radius fracture |
| Ramus of mandible closed fracture of unspecified part |
| Ramus of mandible of unspecified part open fracture |
| Rib fracture |
| Scapula fracture |
| Skull fracture NOS |
| Skull fractured base |
| Smith's fracture |
| Spinal compression fracture |
| Spinal fracture |
| Spinal fracture (excl compression) |
| Spinal fracture NOS |
| Spinal fractures |
| Sternal fracture |
| Stress fracture |
| Subcondylar process of mandible closed fracture |
| Subcondylar process of mandible open fracture |
| Supracondylar fracture of femur, closed |
| Supracondylar fracture of femur, open |
| Symphysis of body of mandible closed fracture |
| Symphysis of body of mandible open fracture |
| Tibia fracture |
| Transcervical fracture, closed |
| Transcervical fracture, open |
| Trimalleolar fracture, closed |
| Trimalleolar fracture, open |
| Ulna fracture |
| Unspecified closed fracture of pelvis |
| Unspecified fracture of ankle, closed |
| Unspecified fracture of ankle, open |
| Unspecified open fracture of pelvis |
| Unspecified site of mandible open fracture |
| Unspecified site of mandible, closed fracture |
| Upper limb fracture NOS |
| Vertebral fracture |
| Wrist fracture |
| Arm fracture |
| Shoulder fracture |
| Fractured coccyx |
| Calcaneus fracture |
| Traumatic fracture |
| Cervical vertebral fracture |
| Lumbar vertebral fracture |
| Thoracic vertebral fracture |
| Cervical vertebral fracture C1 |
| Cervical vertebral fracture C2 |
| Cervical vertebral fracture C4 |
| Cervical vertebral fracture C3 |
| Cervical vertebral fracture C5 |
| Cervical vertebral fracture C6 |
| Cervical vertebral fracture C7 |
| Lumbar vertebral fracture L1 |
| Lumbar vertebral fracture L2 |
| Lumbar vertebral fracture L3 |
| Lumbar vertebral fracture L4 |
| Lumbar vertebral fracture L5 |
| Thoracic vertebral fracture T1 |
| Thoracic vertebral fracture T10 |
| Thoracic vertebral fracture T11 |
| Thoracic vertebral fracture T12 |
| Thoracic vertebral fracture T2 |
| Thoracic vertebral fracture T3 |
| Thoracic vertebral fracture T4 |
| Thoracic vertebral fracture T5 |
| Thoracic vertebral fracture T6 |
| Thoracic vertebral fracture T7 |
| Thoracic vertebral fracture T8 |
| Thoracic vertebral fracture T9 |
| Femoral greater trochanter fracture |
| First rib fracture |
| Second rib fracture |
| Third rib fracture |
| Fourth rib fracture |
| Fifth rib fracture |
| Sixth rib fracture |
| Seventh rib fracture |
| Eighth rib fracture |
| Ninth rib fracture |
| Tenth rib fracture |
| Eleventh rib fracture |
| Twelfth rib fracture |
| Lower limb fracture NOS |
| Fracture of penis |
| Comminuted fracture |
| Fracture displacement |
| Knee fracture |
| Femur fracture subtrochanteric |
| Epiphyseal fracture |
| Fractured radial styloid process |
| Malleolar fracture |
| Fracture laryngotracheal |
| Pelvic fracture |
| Skull fracture |
| Upper limb fracture |
| Lower limb fracture |
| Tooth fracture |
| Condyle fracture of tibia |
| Medial condyle fracture of tibia |
| Lateral condyle fracture of tibia |
| Closed femur fracture |
| Orbital fracture |
| Pelvic insufficiency fracture |
| Growth plate fracture |
| Torus fracture |
| Buckle fracture |
| Bimalleolar fracture |
| Avulsion fracture |
| Impacted fracture |
| Occipital condylar fracture |
| Femoral lesser trochanter fracture |
| Fractured spinous process |
| Trochanteric femoral fracture |
| Periprosthetic fracture |
| Flake fracture |
| Loss of anatomical alignment after fracture reduction |
| Femur shaft fracture |
| Pubis fracture |
| Atypical femur fracture |
| Femur head fracture |
| Atypical femur fracture bilateral |
| Atypical fracture |
| Atypical stress fracture |
| Chance fracture |
| Seat belt fracture |
| Osteochondral fracture |
| Sacroiliac fracture |
| Crescent fracture |
| Limb fracture |
| Spinal fusion fracture |
| Scaphoid bone fracture |
| Tarsal navicular bone fracture |
| Thoracic spine compression fracture |
| Closed fracture |
| Zygomaticomaxillary complex fracture |
| Digital fracture |
| Lumbar spine compression fracture |
| Occult fracture |
| Craniofacial fracture |
| Craniofacial dysjunction fracture |
| Costal cartilage fracture |
| Lisfranc fracture |
| Fatigue fracture |
| Insufficiency fracture |
| Talus fracture |
| Trimalleolar fracture |
| Metaphyseal corner fracture |
| Bucket handle fracture |
| Corner fracture |
| Bone spur fracture |
| Maxillary antrum fracture |
| Paranasal sinus fracture |
| Osteophyte fracture |
| Maisonneuve fracture |
| Stapes fracture |
| Lumbar pedicle fracture |
| Cervical pedicle fracture |
| Thoracic pedicle fracture |
| Cervical spinous process fracture |
| Lumbar spinous process fracture |
| Thoracic spinous process fracture |
| Hairline fracture |
| Ischiopubic fracture |
| Closed fracture of lower end of tibia |
| Galeazzi fracture |
| Le Fort fracture |
| Vertebral end plate fracture |
| Lumbar vertebral end plate fracture |
| Thoracic vertebral end plate fracture |
| Cervical vertebral end plate fracture |
| Combined tibia-fibula fracture |
| Jones fracture |
| Pertrochanteric femoral fracture |
| Intertrochanteric femoral fracture |
| Lumbar transverse process fracture |
| Depressed fracture |
| Combined radius and ulna fracture |
| Impression fracture |
| Parietal fracture |
| Closed arm fracture |
| Capillary fracture |
| Diaphyseal fracture |
| Bone fracture spontaneous |
| Fracture delayed union |
| Fracture due to osteoporosis |
| Fracture nonunion |
| Fracture pathological |
| Fractured delayed union |
| Nonunion of fracture |
| Osteoporosis fracture |
| Osteoporosis with fracture |
| Osteoporotic fracture |
| Pathologic fracture of distal radius and ulna |
| Pathologic fracture of humerus |
| Pathologic fracture of neck of femur |
| Pathologic fracture of other specified part of femur |
| Pathologic fracture of other specified site |
| Pathologic fracture of tibia and fibula |
| Pathologic fracture of vertebrae |
| Pathologic fracture, unspecified site |
| Pathological fracture |
| Spontaneous bone fracture |
| Spontaneous fracture |
| Non-traumatic fracture |
| Pathological fracture of hip |
| Pathologic fracture of femur |
| Impending fracture |
| Fracture blisters |
| Fracture infection |
| Subchondral insufficiency fracture |
| Pseudofracture |
| Fragility fracture |
| Pathological tooth fracture |
| Pathologic fracture of rib |
| Neurogenic fracture |

**Table S2.** All events listed in Individual Case Safety Reports (ICSRs) reporting a fall or fracture with SGLT2 inhibitors in the Eudravigilance from January 1st. 2015 to December 31st. 2022.

|  | **Canagliflozin (N=706)** | **Dapagliflozin (N=884)** |  | **Empagliflozin (N=945)** |  | **Overall (N=2535)** |
| --- | --- | --- | --- | --- | --- | --- |
| **Preferred terms*** |  |  |  |  |  |  |
| Abdominal pain upper | 4 (0.6%) | 2 (0.2%) |  | 0 (0%) |  | 6 (0.2%) |
| Abnormal loss of weight | 1 (0.1%) | 1 (0.1%) |  | 1 (0.1%) |  | 3 (0.1%) |
| Abscess limb | 2 (0.3%) | 0 (0%) |  | 0 (0%) |  | 2 (0.1%) |
| Acidosis | 1 (0.1%) | 0 (0%) |  | 0 (0%) |  | 1 (0.0%) |
| Acute kidney injury | 14 (2.0%) | 3 (0.3%) |  | 4 (0.4%) |  | 21 (0.8%) |
| Adverse drug reaction | 1 (0.1%) | 0 (0%) |  | 0 (0%) |  | 1 (0.0%) |
| Adverse event | 1 (0.1%) | 1 (0.1%) |  | 0 (0%) |  | 2 (0.1%) |
| Albumin urine present | 1 (0.1%) | 0 (0%) |  | 0 (0%) |  | 1 (0.0%) |
| Amputation | 2 (0.3%) | 0 (0%) |  | 0 (0%) |  | 2 (0.1%) |
| Anaemia | 2 (0.3%) | 0 (0%) |  | 0 (0%) |  | 2 (0.1%) |
| Anaemia postoperative | 1 (0.1%) | 0 (0%) |  | 0 (0%) |  | 1 (0.0%) |
| Anal incontinence | 1 (0.1%) | 0 (0%) |  | 0 (0%) |  | 1 (0.0%) |
| Ankle deformity | 1 (0.1%) | 0 (0%) |  | 0 (0%) |  | 1 (0.0%) |
| Ankle fracture | 11 (1.6%) | 8 (0.9%) |  | 7 (0.7%) |  | 26 (1.0%) |
| Aphasia | 1 (0.1%) | 0 (0%) |  | 0 (0%) |  | 1 (0.0%) |
| Aphthous ulcer | 1 (0.1%) | 0 (0%) |  | 0 (0%) |  | 1 (0.0%) |
| Arrhythmia | 1 (0.1%) | 0 (0%) |  | 0 (0%) |  | 1 (0.0%) |
| Arthritis bacterial | 2 (0.3%) | 0 (0%) |  | 0 (0%) |  | 2 (0.1%) |
| Arthropathy | 1 (0.1%) | 2 (0.2%) |  | 0 (0%) |  | 3 (0.1%) |
| Asthenia | 2 (0.3%) | 8 (0.9%) |  | 12 (1.3%) |  | 22 (0.9%) |
| Atrial fibrillation | 1 (0.1%) | 3 (0.3%) |  | 0 (0%) |  | 4 (0.2%) |
| Balance disorder | 2 (0.3%) | 5 (0.6%) |  | 3 (0.3%) |  | 10 (0.4%) |
| Blindness unilateral | 1 (0.1%) | 0 (0%) |  | 1 (0.1%) |  | 2 (0.1%) |
| Blood cholesterol increased | 1 (0.1%) | 6 (0.7%) |  | 1 (0.1%) |  | 8 (0.3%) |
| Blood creatinine increased | 1 (0.1%) | 2 (0.2%) |  | 4 (0.4%) |  | 7 (0.3%) |
| Blood glucose abnormal | 1 (0.1%) | 3 (0.3%) |  | 4 (0.4%) |  | 8 (0.3%) |
| Blood glucose decreased | 3 (0.4%) | 0 (0%) |  | 6 (0.6%) |  | 9 (0.4%) |
| Blood glucose increased | 4 (0.6%) | 16 (1.8%) |  | 14 (1.5%) |  | 34 (1.3%) |
| Blood pressure abnormal | 1 (0.1%) | 0 (0%) |  | 2 (0.2%) |  | 3 (0.1%) |
| Blood pressure decreased | 2 (0.3%) | 4 (0.5%) |  | 0 (0%) |  | 6 (0.2%) |
| Blood pressure increased | 2 (0.3%) | 1 (0.1%) |  | 2 (0.2%) |  | 5 (0.2%) |
| Blood triglycerides increased | 1 (0.1%) | 0 (0%) |  | 0 (0%) |  | 1 (0.0%) |
| Body height decreased | 1 (0.1%) | 2 (0.2%) |  | 0 (0%) |  | 3 (0.1%) |
| Body temperature fluctuation | 1 (0.1%) | 0 (0%) |  | 0 (0%) |  | 1 (0.0%) |
| Bone cancer | 1 (0.1%) | 0 (0%) |  | 0 (0%) |  | 1 (0.0%) |
| Bone marrow oedema syndrome | 1 (0.1%) | 0 (0%) |  | 0 (0%) |  | 1 (0.0%) |
| Bone pain | 1 (0.1%) | 2 (0.2%) |  | 2 (0.2%) |  | 5 (0.2%) |
| Cardiac disorder | 1 (0.1%) | 1 (0.1%) |  | 3 (0.3%) |  | 5 (0.2%) |
| Cardiac failure | 1 (0.1%) | 4 (0.5%) |  | 3 (0.3%) |  | 8 (0.3%) |
| Cardiac failure congestive | 1 (0.1%) | 1 (0.1%) |  | 0 (0%) |  | 2 (0.1%) |
| Cardiac pacemaker insertion | 1 (0.1%) | 0 (0%) |  | 0 (0%) |  | 1 (0.0%) |
| Cardiovascular disorder | 1 (0.1%) | 2 (0.2%) |  | 0 (0%) |  | 3 (0.1%) |
| Carotid artery stenosis | 1 (0.1%) | 1 (0.1%) |  | 0 (0%) |  | 2 (0.1%) |
| Cellulitis | 8 (1.1%) | 0 (0%) |  | 1 (0.1%) |  | 9 (0.4%) |
| Cerebral haemorrhage | 1 (0.1%) | 0 (0%) |  | 4 (0.4%) |  | 5 (0.2%) |
| Cerebrovascular accident | 2 (0.3%) | 5 (0.6%) |  | 5 (0.5%) |  | 12 (0.5%) |
| Chromaturia | 1 (0.1%) | 1 (0.1%) |  | 0 (0%) |  | 2 (0.1%) |
| Clavicle fracture | 1 (0.1%) | 0 (0%) |  | 2 (0.2%) |  | 3 (0.1%) |
| Cognitive disorder | 1 (0.1%) | 0 (0%) |  | 0 (0%) |  | 1 (0.0%) |
| Colon cancer | 1 (0.1%) | 1 (0.1%) |  | 1 (0.1%) |  | 3 (0.1%) |
| Comminuted fracture | 1 (0.1%) | 0 (0%) |  | 0 (0%) |  | 1 (0.0%) |
| Compression fracture | 3 (0.4%) | 2 (0.2%) |  | 0 (0%) |  | 5 (0.2%) |
| Concussion | 1 (0.1%) | 0 (0%) |  | 0 (0%) |  | 1 (0.0%) |
| Condition aggravated | 1 (0.1%) | 1 (0.1%) |  | 0 (0%) |  | 2 (0.1%) |
| Confusional state | 1 (0.1%) | 3 (0.3%) |  | 4 (0.4%) |  | 8 (0.3%) |
| Death | 2 (0.3%) | 1 (0.1%) |  | 1 (0.1%) |  | 4 (0.2%) |
| Decreased appetite | 1 (0.1%) | 9 (1.0%) |  | 5 (0.5%) |  | 15 (0.6%) |
| Dehydration | 7 (1.0%) | 13 (1.5%) |  | 11 (1.2%) |  | 31 (1.2%) |
| Dementia Alzheimer's type | 1 (0.1%) | 0 (0%) |  | 0 (0%) |  | 1 (0.0%) |
| Depressed level of consciousness | 1 (0.1%) | 0 (0%) |  | 0 (0%) |  | 1 (0.0%) |
| Depression suicidal | 1 (0.1%) | 0 (0%) |  | 0 (0%) |  | 1 (0.0%) |
| Dermatitis allergic | 1 (0.1%) | 0 (0%) |  | 0 (0%) |  | 1 (0.0%) |
| Diabetes mellitus inadequate control | 2 (0.3%) | 3 (0.3%) |  | 2 (0.2%) |  | 7 (0.3%) |
| Diabetic foot | 5 (0.7%) | 0 (0%) |  | 0 (0%) |  | 5 (0.2%) |
| Diabetic foot infection | 7 (1.0%) | 0 (0%) |  | 0 (0%) |  | 7 (0.3%) |
| Diabetic gangrene | 1 (0.1%) | 0 (0%) |  | 0 (0%) |  | 1 (0.0%) |
| Diabetic ketoacidosis | 8 (1.1%) | 7 (0.8%) |  | 21 (2.2%) |  | 36 (1.4%) |
| Diabetic neuropathy | 1 (0.1%) | 1 (0.1%) |  | 0 (0%) |  | 2 (0.1%) |
| Diabetic ulcer | 2 (0.3%) | 0 (0%) |  | 0 (0%) |  | 2 (0.1%) |
| Diarrhoea | 1 (0.1%) | 6 (0.7%) |  | 4 (0.4%) |  | 11 (0.4%) |
| Discomfort | 1 (0.1%) | 1 (0.1%) |  | 0 (0%) |  | 2 (0.1%) |
| Disturbance in attention | 1 (0.1%) | 0 (0%) |  | 0 (0%) |  | 1 (0.0%) |
| Dizziness | 8 (1.1%) | 23 (2.6%) |  | 19 (2.0%) |  | 50 (2.0%) |
| Dry gangrene | 2 (0.3%) | 0 (0%) |  | 0 (0%) |  | 2 (0.1%) |
| Dry mouth | 1 (0.1%) | 3 (0.3%) |  | 2 (0.2%) |  | 6 (0.2%) |
| Dysarthria | 1 (0.1%) | 1 (0.1%) |  | 0 (0%) |  | 2 (0.1%) |
| Dyspnoea | 2 (0.3%) | 4 (0.5%) |  | 7 (0.7%) |  | 13 (0.5%) |
| Embolism | 1 (0.1%) | 0 (0%) |  | 0 (0%) |  | 1 (0.0%) |
| Encephalopathy | 2 (0.3%) | 0 (0%) |  | 0 (0%) |  | 2 (0.1%) |
| Enterococcal infection | 1 (0.1%) | 0 (0%) |  | 0 (0%) |  | 1 (0.0%) |
| Erectile dysfunction | 1 (0.1%) | 0 (0%) |  | 0 (0%) |  | 1 (0.0%) |
| Euglycaemic diabetic ketoacidosis | 1 (0.1%) | 2 (0.2%) |  | 12 (1.3%) |  | 15 (0.6%) |
| Extremity necrosis | 2 (0.3%) | 0 (0%) |  | 0 (0%) |  | 2 (0.1%) |
| Eye disorder | 1 (0.1%) | 0 (0%) |  | 0 (0%) |  | 1 (0.0%) |
| Eye infection | 1 (0.1%) | 0 (0%) |  | 0 (0%) |  | 1 (0.0%) |
| Facial spasm | 1 (0.1%) | 0 (0%) |  | 0 (0%) |  | 1 (0.0%) |
| Fall | 100 (14.2%) | 101 (11.4%) |  | 132 (14.0%) |  | 333 (13.1%) |
| Fatigue | 2 (0.3%) | 3 (0.3%) |  | 5 (0.5%) |  | 10 (0.4%) |
| Feeling abnormal | 1 (0.1%) | 4 (0.5%) |  | 4 (0.4%) |  | 9 (0.4%) |
| Femoral neck fracture | 2 (0.3%) | 2 (0.2%) |  | 4 (0.4%) |  | 8 (0.3%) |
| Femur fracture | 8 (1.1%) | 10 (1.1%) |  | 9 (1.0%) |  | 27 (1.1%) |
| Fibula fracture | 2 (0.3%) | 0 (0%) |  | 1 (0.1%) |  | 3 (0.1%) |
| Fluid retention | 1 (0.1%) | 0 (0%) |  | 0 (0%) |  | 1 (0.0%) |
| Foot amputation | 2 (0.3%) | 0 (0%) |  | 0 (0%) |  | 2 (0.1%) |
| Foot fracture | 27 (3.8%) | 8 (0.9%) |  | 5 (0.5%) |  | 40 (1.6%) |
| Forearm fracture | 1 (0.1%) | 0 (0%) |  | 2 (0.2%) |  | 3 (0.1%) |
| Fournier's gangrene | 2 (0.3%) | 0 (0%) |  | 2 (0.2%) |  | 4 (0.2%) |
| Fracture | 14 (2.0%) | 6 (0.7%) |  | 4 (0.4%) |  | 24 (0.9%) |
| Fracture displacement | 1 (0.1%) | 0 (0%) |  | 0 (0%) |  | 1 (0.0%) |
| Fracture nonunion | 2 (0.3%) | 0 (0%) |  | 0 (0%) |  | 2 (0.1%) |
| Fungal infection | 5 (0.7%) | 4 (0.5%) |  | 3 (0.3%) |  | 12 (0.5%) |
| Fungal skin infection | 1 (0.1%) | 0 (0%) |  | 1 (0.1%) |  | 2 (0.1%) |
| Gait disturbance | 3 (0.4%) | 6 (0.7%) |  | 5 (0.5%) |  | 14 (0.6%) |
| Gait inability | 2 (0.3%) | 0 (0%) |  | 1 (0.1%) |  | 3 (0.1%) |
| Gallbladder disorder | 1 (0.1%) | 0 (0%) |  | 0 (0%) |  | 1 (0.0%) |
| Gangrene | 8 (1.1%) | 0 (0%) |  | 0 (0%) |  | 8 (0.3%) |
| Gas gangrene | 1 (0.1%) | 0 (0%) |  | 0 (0%) |  | 1 (0.0%) |
| Glucose urine present | 1 (0.1%) | 0 (0%) |  | 1 (0.1%) |  | 2 (0.1%) |
| Glycosylated haemoglobin increased | 2 (0.3%) | 3 (0.3%) |  | 2 (0.2%) |  | 7 (0.3%) |
| Haematemesis | 1 (0.1%) | 0 (0%) |  | 1 (0.1%) |  | 2 (0.1%) |
| Haemorrhage | 1 (0.1%) | 0 (0%) |  | 0 (0%) |  | 1 (0.0%) |
| Haemothorax | 1 (0.1%) | 0 (0%) |  | 0 (0%) |  | 1 (0.0%) |
| Hand fracture | 1 (0.1%) | 1 (0.1%) |  | 1 (0.1%) |  | 3 (0.1%) |
| Head injury | 1 (0.1%) | 5 (0.6%) |  | 4 (0.4%) |  | 10 (0.4%) |
| Hepatic function abnormal | 2 (0.3%) | 1 (0.1%) |  | 0 (0%) |  | 3 (0.1%) |
| Hepatic infection | 1 (0.1%) | 0 (0%) |  | 0 (0%) |  | 1 (0.0%) |
| Herpes zoster | 1 (0.1%) | 0 (0%) |  | 0 (0%) |  | 1 (0.0%) |
| Hip fracture | 10 (1.4%) | 4 (0.5%) |  | 18 (1.9%) |  | 32 (1.3%) |
| Humerus fracture | 6 (0.8%) | 3 (0.3%) |  | 4 (0.4%) |  | 13 (0.5%) |
| Hyperchlorhydria | 1 (0.1%) | 0 (0%) |  | 0 (0%) |  | 1 (0.0%) |
| Hyperglycaemic hyperosmolar nonketotic syndrome | 1 (0.1%) | 2 (0.2%) |  | 0 (0%) |  | 3 (0.1%) |
| Hyperhidrosis | 1 (0.1%) | 4 (0.5%) |  | 1 (0.1%) |  | 6 (0.2%) |
| Hyperkalaemia | 3 (0.4%) | 1 (0.1%) |  | 2 (0.2%) |  | 6 (0.2%) |
| Hypersensitivity | 1 (0.1%) | 1 (0.1%) |  | 0 (0%) |  | 2 (0.1%) |
| Hypersomnia | 1 (0.1%) | 0 (0%) |  | 0 (0%) |  | 1 (0.0%) |
| Hypertension | 1 (0.1%) | 2 (0.2%) |  | 4 (0.4%) |  | 7 (0.3%) |
| Hypoacusis | 1 (0.1%) | 1 (0.1%) |  | 1 (0.1%) |  | 3 (0.1%) |
| Hypoaesthesia | 1 (0.1%) | 2 (0.2%) |  | 2 (0.2%) |  | 5 (0.2%) |
| Hypoglycaemia | 2 (0.3%) | 6 (0.7%) |  | 8 (0.8%) |  | 16 (0.6%) |
| Hypotension | 4 (0.6%) | 12 (1.4%) |  | 8 (0.8%) |  | 24 (0.9%) |
| Hypothermia | 1 (0.1%) | 0 (0%) |  | 0 (0%) |  | 1 (0.0%) |
| Hypovolaemic shock | 1 (0.1%) | 0 (0%) |  | 0 (0%) |  | 1 (0.0%) |
| Illness | 1 (0.1%) | 1 (0.1%) |  | 1 (0.1%) |  | 3 (0.1%) |
| Impaired healing | 10 (1.4%) | 0 (0%) |  | 1 (0.1%) |  | 11 (0.4%) |
| Impaired work ability | 1 (0.1%) | 0 (0%) |  | 0 (0%) |  | 1 (0.0%) |
| Inappropriate schedule of product administration | 6 (0.8%) | 0 (0%) |  | 2 (0.2%) |  | 8 (0.3%) |
| Incorrect dose administered | 4 (0.6%) | 0 (0%) |  | 1 (0.1%) |  | 5 (0.2%) |
| Infection | 3 (0.4%) | 1 (0.1%) |  | 1 (0.1%) |  | 5 (0.2%) |
| Injury | 4 (0.6%) | 2 (0.2%) |  | 0 (0%) |  | 6 (0.2%) |
| International normalised ratio increased | 1 (0.1%) | 0 (0%) |  | 0 (0%) |  | 1 (0.0%) |
| Intracranial haematoma | 1 (0.1%) | 0 (0%) |  | 0 (0%) |  | 1 (0.0%) |
| Joint dislocation | 3 (0.4%) | 1 (0.1%) |  | 2 (0.2%) |  | 6 (0.2%) |
| Joint injury | 3 (0.4%) | 1 (0.1%) |  | 1 (0.1%) |  | 5 (0.2%) |
| Ketoacidosis | 1 (0.1%) | 3 (0.3%) |  | 10 (1.1%) |  | 14 (0.5%) |
| Leg amputation | 8 (1.1%) | 1 (0.1%) |  | 0 (0%) |  | 9 (0.4%) |
| Libido decreased | 1 (0.1%) | 0 (0%) |  | 0 (0%) |  | 1 (0.0%) |
| Ligament disorder | 1 (0.1%) | 0 (0%) |  | 0 (0%) |  | 1 (0.0%) |
| Ligament sprain | 1 (0.1%) | 3 (0.3%) |  | 0 (0%) |  | 4 (0.2%) |
| Limb amputation | 8 (1.1%) | 0 (0%) |  | 0 (0%) |  | 8 (0.3%) |
| Limb injury | 1 (0.1%) | 5 (0.6%) |  | 4 (0.4%) |  | 10 (0.4%) |
| Limb traumatic amputation | 1 (0.1%) | 0 (0%) |  | 0 (0%) |  | 1 (0.0%) |
| Lisfranc fracture | 1 (0.1%) | 0 (0%) |  | 0 (0%) |  | 1 (0.0%) |
| Liver function test increased | 1 (0.1%) | 1 (0.1%) |  | 0 (0%) |  | 2 (0.1%) |
| Liver injury | 1 (0.1%) | 0 (0%) |  | 0 (0%) |  | 1 (0.0%) |
| Localised infection | 2 (0.3%) | 1 (0.1%) |  | 1 (0.1%) |  | 4 (0.2%) |
| Loss of consciousness | 4 (0.6%) | 4 (0.5%) |  | 7 (0.7%) |  | 15 (0.6%) |
| Loss of personal independence in daily activities | 1 (0.1%) | 0 (0%) |  | 1 (0.1%) |  | 2 (0.1%) |
| Lower limb fracture | 6 (0.8%) | 9 (1.0%) |  | 8 (0.8%) |  | 23 (0.9%) |
| Lumbosacral radiculoplexus neuropathy | 1 (0.1%) | 0 (0%) |  | 0 (0%) |  | 1 (0.0%) |
| Malaise | 1 (0.1%) | 11 (1.2%) |  | 7 (0.7%) |  | 19 (0.7%) |
| Medication error | 1 (0.1%) | 1 (0.1%) |  | 0 (0%) |  | 2 (0.1%) |
| Mental impairment | 1 (0.1%) | 0 (0%) |  | 0 (0%) |  | 1 (0.0%) |
| Motion sickness | 1 (0.1%) | 0 (0%) |  | 0 (0%) |  | 1 (0.0%) |
| Multiple injuries | 1 (0.1%) | 0 (0%) |  | 0 (0%) |  | 1 (0.0%) |
| Muscle contracture | 1 (0.1%) | 0 (0%) |  | 0 (0%) |  | 1 (0.0%) |
| Muscle spasms | 1 (0.1%) | 0 (0%) |  | 1 (0.1%) |  | 2 (0.1%) |
| Muscular weakness | 1 (0.1%) | 3 (0.3%) |  | 1 (0.1%) |  | 5 (0.2%) |
| Myocardial infarction | 3 (0.4%) | 4 (0.5%) |  | 3 (0.3%) |  | 10 (0.4%) |
| Nausea | 3 (0.4%) | 4 (0.5%) |  | 8 (0.8%) |  | 15 (0.6%) |
| Necrosis | 2 (0.3%) | 0 (0%) |  | 1 (0.1%) |  | 3 (0.1%) |
| Necrotising fasciitis | 1 (0.1%) | 1 (0.1%) |  | 0 (0%) |  | 2 (0.1%) |
| Nervousness | 1 (0.1%) | 0 (0%) |  | 0 (0%) |  | 1 (0.0%) |
| Neuropathic arthropathy | 2 (0.3%) | 0 (0%) |  | 1 (0.1%) |  | 3 (0.1%) |
| Neuropathy peripheral | 1 (0.1%) | 1 (0.1%) |  | 2 (0.2%) |  | 4 (0.2%) |
| Nocturia | 3 (0.4%) | 1 (0.1%) |  | 2 (0.2%) |  | 6 (0.2%) |
| Ocular dysmetria | 1 (0.1%) | 0 (0%) |  | 0 (0%) |  | 1 (0.0%) |
| Oesophagitis | 1 (0.1%) | 0 (0%) |  | 0 (0%) |  | 1 (0.0%) |
| Off label use | 6 (0.8%) | 12 (1.4%) |  | 3 (0.3%) |  | 21 (0.8%) |
| Open fracture | 1 (0.1%) | 0 (0%) |  | 0 (0%) |  | 1 (0.0%) |
| Orthostatic hypotension | 1 (0.1%) | 3 (0.3%) |  | 2 (0.2%) |  | 6 (0.2%) |
| Osteoarthritis | 1 (0.1%) | 3 (0.3%) |  | 0 (0%) |  | 4 (0.2%) |
| Osteomyelitis | 16 (2.3%) | 0 (0%) |  | 1 (0.1%) |  | 17 (0.7%) |
| Osteomyelitis acute | 3 (0.4%) | 0 (0%) |  | 0 (0%) |  | 3 (0.1%) |
| Osteomyelitis chronic | 4 (0.6%) | 0 (0%) |  | 0 (0%) |  | 4 (0.2%) |
| Osteopenia | 1 (0.1%) | 0 (0%) |  | 0 (0%) |  | 1 (0.0%) |
| Osteoporosis | 1 (0.1%) | 1 (0.1%) |  | 0 (0%) |  | 2 (0.1%) |
| Pain | 3 (0.4%) | 6 (0.7%) |  | 3 (0.3%) |  | 12 (0.5%) |
| Pain in extremity | 3 (0.4%) | 1 (0.1%) |  | 4 (0.4%) |  | 8 (0.3%) |
| Pancreatitis acute | 1 (0.1%) | 0 (0%) |  | 0 (0%) |  | 1 (0.0%) |
| Patella fracture | 3 (0.4%) | 0 (0%) |  | 0 (0%) |  | 3 (0.1%) |
| Peripheral arterial occlusive disease | 1 (0.1%) | 0 (0%) |  | 1 (0.1%) |  | 2 (0.1%) |
| Peripheral ischaemia | 1 (0.1%) | 0 (0%) |  | 0 (0%) |  | 1 (0.0%) |
| Peripheral swelling | 2 (0.3%) | 0 (0%) |  | 1 (0.1%) |  | 3 (0.1%) |
| Physical deconditioning | 1 (0.1%) | 0 (0%) |  | 0 (0%) |  | 1 (0.0%) |
| Pneumonia | 1 (0.1%) | 5 (0.6%) |  | 6 (0.6%) |  | 12 (0.5%) |
| Pollakiuria | 4 (0.6%) | 5 (0.6%) |  | 5 (0.5%) |  | 14 (0.6%) |
| Postoperative wound infection | 1 (0.1%) | 0 (0%) |  | 0 (0%) |  | 1 (0.0%) |
| Product use in unapproved indication | 1 (0.1%) | 2 (0.2%) |  | 0 (0%) |  | 3 (0.1%) |
| Protein urine present | 1 (0.1%) | 0 (0%) |  | 0 (0%) |  | 1 (0.0%) |
| Pruritus | 1 (0.1%) | 1 (0.1%) |  | 1 (0.1%) |  | 3 (0.1%) |
| Pulmonary embolism | 1 (0.1%) | 0 (0%) |  | 0 (0%) |  | 1 (0.0%) |
| Pulmonary function test decreased | 1 (0.1%) | 0 (0%) |  | 0 (0%) |  | 1 (0.0%) |
| Pyrexia | 1 (0.1%) | 0 (0%) |  | 3 (0.3%) |  | 4 (0.2%) |
| Radius fracture | 1 (0.1%) | 0 (0%) |  | 2 (0.2%) |  | 3 (0.1%) |
| Rash | 3 (0.4%) | 0 (0%) |  | 1 (0.1%) |  | 4 (0.2%) |
| Rash pruritic | 1 (0.1%) | 2 (0.2%) |  | 0 (0%) |  | 3 (0.1%) |
| Renal failure | 2 (0.3%) | 3 (0.3%) |  | 3 (0.3%) |  | 8 (0.3%) |
| Renal injury | 1 (0.1%) | 1 (0.1%) |  | 0 (0%) |  | 2 (0.1%) |
| Renal pain | 1 (0.1%) | 0 (0%) |  | 1 (0.1%) |  | 2 (0.1%) |
| Respiratory failure | 2 (0.3%) | 0 (0%) |  | 1 (0.1%) |  | 3 (0.1%) |
| Rib fracture | 6 (0.8%) | 5 (0.6%) |  | 6 (0.6%) |  | 17 (0.7%) |
| Road traffic accident | 8 (1.1%) | 2 (0.2%) |  | 2 (0.2%) |  | 12 (0.5%) |
| Scapula fracture | 1 (0.1%) | 0 (0%) |  | 1 (0.1%) |  | 2 (0.1%) |
| Scar | 1 (0.1%) | 0 (0%) |  | 0 (0%) |  | 1 (0.0%) |
| Seizure | 1 (0.1%) | 0 (0%) |  | 1 (0.1%) |  | 2 (0.1%) |
| Sepsis | 7 (1.0%) | 0 (0%) |  | 2 (0.2%) |  | 9 (0.4%) |
| Shoulder arthroplasty | 1 (0.1%) | 0 (0%) |  | 0 (0%) |  | 1 (0.0%) |
| Skin laceration | 2 (0.3%) | 0 (0%) |  | 0 (0%) |  | 2 (0.1%) |
| Skin ulcer | 2 (0.3%) | 0 (0%) |  | 0 (0%) |  | 2 (0.1%) |
| Soft tissue infection | 1 (0.1%) | 0 (0%) |  | 0 (0%) |  | 1 (0.0%) |
| Soft tissue necrosis | 1 (0.1%) | 0 (0%) |  | 0 (0%) |  | 1 (0.0%) |
| Spinal compression fracture | 6 (0.8%) | 4 (0.5%) |  | 3 (0.3%) |  | 13 (0.5%) |
| Spinal fracture | 2 (0.3%) | 0 (0%) |  | 1 (0.1%) |  | 3 (0.1%) |
| Spinal ligament ossification | 1 (0.1%) | 0 (0%) |  | 0 (0%) |  | 1 (0.0%) |
| Spinal osteoarthritis | 1 (0.1%) | 0 (0%) |  | 0 (0%) |  | 1 (0.0%) |
| Staphylococcal infection | 1 (0.1%) | 0 (0%) |  | 0 (0%) |  | 1 (0.0%) |
| Subdural hygroma | 2 (0.3%) | 0 (0%) |  | 0 (0%) |  | 2 (0.1%) |
| Sudden hearing loss | 1 (0.1%) | 0 (0%) |  | 0 (0%) |  | 1 (0.0%) |
| Suicidal ideation | 1 (0.1%) | 0 (0%) |  | 0 (0%) |  | 1 (0.0%) |
| Tachycardia | 1 (0.1%) | 0 (0%) |  | 2 (0.2%) |  | 3 (0.1%) |
| Tendon disorder | 1 (0.1%) | 0 (0%) |  | 0 (0%) |  | 1 (0.0%) |
| Tendon injury | 1 (0.1%) | 0 (0%) |  | 0 (0%) |  | 1 (0.0%) |
| Tendon rupture | 1 (0.1%) | 0 (0%) |  | 0 (0%) |  | 1 (0.0%) |
| Tension headache | 1 (0.1%) | 0 (0%) |  | 0 (0%) |  | 1 (0.0%) |
| Thirst | 2 (0.3%) | 1 (0.1%) |  | 1 (0.1%) |  | 4 (0.2%) |
| Tibia fracture | 6 (0.8%) | 1 (0.1%) |  | 1 (0.1%) |  | 8 (0.3%) |
| Toe amputation | 10 (1.4%) | 0 (0%) |  | 0 (0%) |  | 10 (0.4%) |
| Transient ischaemic attack | 2 (0.3%) | 1 (0.1%) |  | 1 (0.1%) |  | 4 (0.2%) |
| Tremor | 2 (0.3%) | 0 (0%) |  | 3 (0.3%) |  | 5 (0.2%) |
| Ulcer | 1 (0.1%) | 0 (0%) |  | 1 (0.1%) |  | 2 (0.1%) |
| Upper limb fracture | 10 (1.4%) | 7 (0.8%) |  | 6 (0.6%) |  | 23 (0.9%) |
| Upper respiratory tract infection | 2 (0.3%) | 1 (0.1%) |  | 0 (0%) |  | 3 (0.1%) |
| Urinary incontinence | 3 (0.4%) | 1 (0.1%) |  | 4 (0.4%) |  | 8 (0.3%) |
| Urinary retention | 1 (0.1%) | 1 (0.1%) |  | 1 (0.1%) |  | 3 (0.1%) |
| Urinary tract infection | 10 (1.4%) | 4 (0.5%) |  | 7 (0.7%) |  | 21 (0.8%) |
| Urine ketone body present | 2 (0.3%) | 0 (0%) |  | 2 (0.2%) |  | 4 (0.2%) |
| Urosepsis | 1 (0.1%) | 1 (0.1%) |  | 2 (0.2%) |  | 4 (0.2%) |
| Urticaria | 2 (0.3%) | 1 (0.1%) |  | 0 (0%) |  | 3 (0.1%) |
| Vertigo | 1 (0.1%) | 0 (0%) |  | 3 (0.3%) |  | 4 (0.2%) |
| Visual impairment | 2 (0.3%) | 1 (0.1%) |  | 2 (0.2%) |  | 5 (0.2%) |
| Vomiting | 1 (0.1%) | 0 (0%) |  | 5 (0.5%) |  | 6 (0.2%) |
| Weight decreased | 4 (0.6%) | 20 (2.3%) |  | 10 (1.1%) |  | 34 (1.3%) |
| Weight increased | 1 (0.1%) | 7 (0.8%) |  | 2 (0.2%) |  | 10 (0.4%) |
| Wound | 5 (0.7%) | 2 (0.2%) |  | 2 (0.2%) |  | 9 (0.4%) |
| Wound dehiscence | 3 (0.4%) | 0 (0%) |  | 0 (0%) |  | 3 (0.1%) |
| Wound infection | 1 (0.1%) | 0 (0%) |  | 0 (0%) |  | 1 (0.0%) |
| Wrist fracture | 4 (0.6%) | 3 (0.3%) |  | 2 (0.2%) |  | 9 (0.4%) |
| Abdominal discomfort | 0 (0%) | 3 (0.3%) |  | 2 (0.2%) |  | 5 (0.2%) |
| Abdominal pain | 0 (0%) | 1 (0.1%) |  | 4 (0.4%) |  | 5 (0.2%) |
| Abdominal tenderness | 0 (0%) | 1 (0.1%) |  | 1 (0.1%) |  | 2 (0.1%) |
| Abnormal behaviour | 0 (0%) | 1 (0.1%) |  | 0 (0%) |  | 1 (0.0%) |
| Abnormal faeces | 0 (0%) | 1 (0.1%) |  | 0 (0%) |  | 1 (0.0%) |
| Accident | 0 (0%) | 2 (0.2%) |  | 0 (0%) |  | 2 (0.1%) |
| Accident at home | 0 (0%) | 1 (0.1%) |  | 0 (0%) |  | 1 (0.0%) |
| Acute myocardial infarction | 0 (0%) | 1 (0.1%) |  | 1 (0.1%) |  | 2 (0.1%) |
| Altered state of consciousness | 0 (0%) | 4 (0.5%) |  | 0 (0%) |  | 4 (0.2%) |
| Anxiety | 0 (0%) | 1 (0.1%) |  | 2 (0.2%) |  | 3 (0.1%) |
| Apathy | 0 (0%) | 1 (0.1%) |  | 0 (0%) |  | 1 (0.0%) |
| Arthralgia | 0 (0%) | 8 (0.9%) |  | 8 (0.8%) |  | 16 (0.6%) |
| Arthritis | 0 (0%) | 2 (0.2%) |  | 0 (0%) |  | 2 (0.1%) |
| Asthma | 0 (0%) | 1 (0.1%) |  | 0 (0%) |  | 1 (0.0%) |
| Back injury | 0 (0%) | 4 (0.5%) |  | 0 (0%) |  | 4 (0.2%) |
| Back pain | 0 (0%) | 2 (0.2%) |  | 3 (0.3%) |  | 5 (0.2%) |
| Balanoposthitis | 0 (0%) | 2 (0.2%) |  | 0 (0%) |  | 2 (0.1%) |
| Bifascicular block | 0 (0%) | 1 (0.1%) |  | 0 (0%) |  | 1 (0.0%) |
| Bladder cancer | 0 (0%) | 1 (0.1%) |  | 0 (0%) |  | 1 (0.0%) |
| Bladder injury | 0 (0%) | 1 (0.1%) |  | 0 (0%) |  | 1 (0.0%) |
| Blindness | 0 (0%) | 2 (0.2%) |  | 1 (0.1%) |  | 3 (0.1%) |
| Blood creatine phosphokinase increased | 0 (0%) | 1 (0.1%) |  | 0 (0%) |  | 1 (0.0%) |
| Blood ketone body increased | 0 (0%) | 1 (0.1%) |  | 1 (0.1%) |  | 2 (0.1%) |
| Bone contusion | 0 (0%) | 1 (0.1%) |  | 1 (0.1%) |  | 2 (0.1%) |
| Bone density decreased | 0 (0%) | 1 (0.1%) |  | 0 (0%) |  | 1 (0.0%) |
| Bradycardia | 0 (0%) | 1 (0.1%) |  | 1 (0.1%) |  | 2 (0.1%) |
| Brain cancer metastatic | 0 (0%) | 1 (0.1%) |  | 0 (0%) |  | 1 (0.0%) |
| Brain stem haemorrhage | 0 (0%) | 1 (0.1%) |  | 0 (0%) |  | 1 (0.0%) |
| Breast enlargement | 0 (0%) | 1 (0.1%) |  | 0 (0%) |  | 1 (0.0%) |
| Bronchitis | 0 (0%) | 1 (0.1%) |  | 1 (0.1%) |  | 2 (0.1%) |
| Cardiac flutter | 0 (0%) | 1 (0.1%) |  | 0 (0%) |  | 1 (0.0%) |
| Carotid artery occlusion | 0 (0%) | 1 (0.1%) |  | 0 (0%) |  | 1 (0.0%) |
| Carotid sinus syndrome | 0 (0%) | 1 (0.1%) |  | 0 (0%) |  | 1 (0.0%) |
| Cataract | 0 (0%) | 1 (0.1%) |  | 1 (0.1%) |  | 2 (0.1%) |
| Cerebellar infarction | 0 (0%) | 1 (0.1%) |  | 1 (0.1%) |  | 2 (0.1%) |
| Cerebral cyst | 0 (0%) | 1 (0.1%) |  | 0 (0%) |  | 1 (0.0%) |
| Cerebral disorder | 0 (0%) | 1 (0.1%) |  | 0 (0%) |  | 1 (0.0%) |
| Cerebral infarction | 0 (0%) | 2 (0.2%) |  | 2 (0.2%) |  | 4 (0.2%) |
| Cerebrovascular disorder | 0 (0%) | 1 (0.1%) |  | 0 (0%) |  | 1 (0.0%) |
| Chest pain | 0 (0%) | 1 (0.1%) |  | 2 (0.2%) |  | 3 (0.1%) |
| Choking | 0 (0%) | 1 (0.1%) |  | 0 (0%) |  | 1 (0.0%) |
| Cholecystitis acute | 0 (0%) | 1 (0.1%) |  | 0 (0%) |  | 1 (0.0%) |
| Chronic kidney disease | 0 (0%) | 3 (0.3%) |  | 0 (0%) |  | 3 (0.1%) |
| Coma | 0 (0%) | 1 (0.1%) |  | 4 (0.4%) |  | 5 (0.2%) |
| Complication associated with device | 0 (0%) | 1 (0.1%) |  | 0 (0%) |  | 1 (0.0%) |
| Constipation | 0 (0%) | 1 (0.1%) |  | 0 (0%) |  | 1 (0.0%) |
| Contusion | 0 (0%) | 8 (0.9%) |  | 4 (0.4%) |  | 12 (0.5%) |
| Craniocerebral injury | 0 (0%) | 1 (0.1%) |  | 0 (0%) |  | 1 (0.0%) |
| Cystitis | 0 (0%) | 2 (0.2%) |  | 1 (0.1%) |  | 3 (0.1%) |
| Delirium | 0 (0%) | 1 (0.1%) |  | 3 (0.3%) |  | 4 (0.2%) |
| Dementia with Lewy bodies | 0 (0%) | 1 (0.1%) |  | 0 (0%) |  | 1 (0.0%) |
| Depressed mood | 0 (0%) | 1 (0.1%) |  | 1 (0.1%) |  | 2 (0.1%) |
| Device delivery system issue | 0 (0%) | 2 (0.2%) |  | 0 (0%) |  | 2 (0.1%) |
| Device issue | 0 (0%) | 3 (0.3%) |  | 0 (0%) |  | 3 (0.1%) |
| Device leakage | 0 (0%) | 2 (0.2%) |  | 0 (0%) |  | 2 (0.1%) |
| Device related infection | 0 (0%) | 1 (0.1%) |  | 0 (0%) |  | 1 (0.0%) |
| Diabetes mellitus | 0 (0%) | 5 (0.6%) |  | 1 (0.1%) |  | 6 (0.2%) |
| Diabetic coma | 0 (0%) | 1 (0.1%) |  | 1 (0.1%) |  | 2 (0.1%) |
| Diabetic ketosis | 0 (0%) | 1 (0.1%) |  | 0 (0%) |  | 1 (0.0%) |
| Diverticulitis | 0 (0%) | 1 (0.1%) |  | 0 (0%) |  | 1 (0.0%) |
| Dizziness postural | 0 (0%) | 1 (0.1%) |  | 0 (0%) |  | 1 (0.0%) |
| Drug hypersensitivity | 0 (0%) | 2 (0.2%) |  | 0 (0%) |  | 2 (0.1%) |
| Drug ineffective | 0 (0%) | 6 (0.7%) |  | 5 (0.5%) |  | 11 (0.4%) |
| Drug interaction | 0 (0%) | 2 (0.2%) |  | 0 (0%) |  | 2 (0.1%) |
| Dyskinesia | 0 (0%) | 1 (0.1%) |  | 1 (0.1%) |  | 2 (0.1%) |
| Dyspepsia | 0 (0%) | 2 (0.2%) |  | 1 (0.1%) |  | 3 (0.1%) |
| Dysphagia | 0 (0%) | 2 (0.2%) |  | 0 (0%) |  | 2 (0.1%) |
| Dysphonia | 0 (0%) | 1 (0.1%) |  | 0 (0%) |  | 1 (0.0%) |
| Dysstasia | 0 (0%) | 1 (0.1%) |  | 1 (0.1%) |  | 2 (0.1%) |
| Dysuria | 0 (0%) | 1 (0.1%) |  | 2 (0.2%) |  | 3 (0.1%) |
| Ear disorder | 0 (0%) | 1 (0.1%) |  | 0 (0%) |  | 1 (0.0%) |
| Ear injury | 0 (0%) | 1 (0.1%) |  | 0 (0%) |  | 1 (0.0%) |
| Ear pain | 0 (0%) | 1 (0.1%) |  | 0 (0%) |  | 1 (0.0%) |
| Eating disorder | 0 (0%) | 1 (0.1%) |  | 0 (0%) |  | 1 (0.0%) |
| Electrocardiogram change | 0 (0%) | 1 (0.1%) |  | 0 (0%) |  | 1 (0.0%) |
| Electrocardiogram ST-T change | 0 (0%) | 1 (0.1%) |  | 0 (0%) |  | 1 (0.0%) |
| End stage renal disease | 0 (0%) | 1 (0.1%) |  | 0 (0%) |  | 1 (0.0%) |
| Escherichia sepsis | 0 (0%) | 1 (0.1%) |  | 0 (0%) |  | 1 (0.0%) |
| Escherichia urinary tract infection | 0 (0%) | 2 (0.2%) |  | 0 (0%) |  | 2 (0.1%) |
| Essential hypertension | 0 (0%) | 1 (0.1%) |  | 0 (0%) |  | 1 (0.0%) |
| Face injury | 0 (0%) | 1 (0.1%) |  | 0 (0%) |  | 1 (0.0%) |
| Facial bones fracture | 0 (0%) | 1 (0.1%) |  | 1 (0.1%) |  | 2 (0.1%) |
| Faecaloma | 0 (0%) | 1 (0.1%) |  | 0 (0%) |  | 1 (0.0%) |
| Feeding disorder | 0 (0%) | 1 (0.1%) |  | 0 (0%) |  | 1 (0.0%) |
| Feeling drunk | 0 (0%) | 1 (0.1%) |  | 0 (0%) |  | 1 (0.0%) |
| Fibrin D dimer increased | 0 (0%) | 1 (0.1%) |  | 0 (0%) |  | 1 (0.0%) |
| Flatulence | 0 (0%) | 1 (0.1%) |  | 1 (0.1%) |  | 2 (0.1%) |
| Fluid intake reduced | 0 (0%) | 2 (0.2%) |  | 0 (0%) |  | 2 (0.1%) |
| Food craving | 0 (0%) | 1 (0.1%) |  | 0 (0%) |  | 1 (0.0%) |
| Furuncle | 0 (0%) | 1 (0.1%) |  | 0 (0%) |  | 1 (0.0%) |
| Gastric cancer | 0 (0%) | 1 (0.1%) |  | 0 (0%) |  | 1 (0.0%) |
| Gastritis | 0 (0%) | 1 (0.1%) |  | 0 (0%) |  | 1 (0.0%) |
| Gastrooesophageal reflux disease | 0 (0%) | 3 (0.3%) |  | 1 (0.1%) |  | 4 (0.2%) |
| General physical health deterioration | 0 (0%) | 2 (0.2%) |  | 5 (0.5%) |  | 7 (0.3%) |
| Genital candidiasis | 0 (0%) | 1 (0.1%) |  | 0 (0%) |  | 1 (0.0%) |
| Genital infection fungal | 0 (0%) | 1 (0.1%) |  | 4 (0.4%) |  | 5 (0.2%) |
| Glycosylated haemoglobin abnormal | 0 (0%) | 1 (0.1%) |  | 2 (0.2%) |  | 3 (0.1%) |
| Glycosylated haemoglobin decreased | 0 (0%) | 1 (0.1%) |  | 0 (0%) |  | 1 (0.0%) |
| Haematuria | 0 (0%) | 1 (0.1%) |  | 1 (0.1%) |  | 2 (0.1%) |
| Hallucination | 0 (0%) | 2 (0.2%) |  | 0 (0%) |  | 2 (0.1%) |
| Headache | 0 (0%) | 5 (0.6%) |  | 2 (0.2%) |  | 7 (0.3%) |
| Heart rate increased | 0 (0%) | 2 (0.2%) |  | 2 (0.2%) |  | 4 (0.2%) |
| Hemiplegia | 0 (0%) | 1 (0.1%) |  | 1 (0.1%) |  | 2 (0.1%) |
| Hernia | 0 (0%) | 1 (0.1%) |  | 0 (0%) |  | 1 (0.0%) |
| Hypercholesterolaemia | 0 (0%) | 1 (0.1%) |  | 0 (0%) |  | 1 (0.0%) |
| Hyperglycaemia | 0 (0%) | 1 (0.1%) |  | 2 (0.2%) |  | 3 (0.1%) |
| Hyperglycaemic unconsciousness | 0 (0%) | 1 (0.1%) |  | 0 (0%) |  | 1 (0.0%) |
| Hypernatraemia | 0 (0%) | 3 (0.3%) |  | 0 (0%) |  | 3 (0.1%) |
| Hypoglycaemic coma | 0 (0%) | 1 (0.1%) |  | 0 (0%) |  | 1 (0.0%) |
| Hypokalaemia | 0 (0%) | 1 (0.1%) |  | 1 (0.1%) |  | 2 (0.1%) |
| Hypometabolism | 0 (0%) | 1 (0.1%) |  | 0 (0%) |  | 1 (0.0%) |
| Hyponatraemia | 0 (0%) | 1 (0.1%) |  | 2 (0.2%) |  | 3 (0.1%) |
| Hypophagia | 0 (0%) | 2 (0.2%) |  | 1 (0.1%) |  | 3 (0.1%) |
| Hypothyroidism | 0 (0%) | 1 (0.1%) |  | 1 (0.1%) |  | 2 (0.1%) |
| Immunodeficiency | 0 (0%) | 1 (0.1%) |  | 0 (0%) |  | 1 (0.0%) |
| Incisional hernia | 0 (0%) | 1 (0.1%) |  | 0 (0%) |  | 1 (0.0%) |
| Incontinence | 0 (0%) | 1 (0.1%) |  | 0 (0%) |  | 1 (0.0%) |
| Incorrect dose administered by device | 0 (0%) | 4 (0.5%) |  | 0 (0%) |  | 4 (0.2%) |
| Infarction | 0 (0%) | 1 (0.1%) |  | 2 (0.2%) |  | 3 (0.1%) |
| Infective exacerbation of chronic obstructive airways disease | 0 (0%) | 1 (0.1%) |  | 0 (0%) |  | 1 (0.0%) |
| Influenza | 0 (0%) | 1 (0.1%) |  | 0 (0%) |  | 1 (0.0%) |
| Ingrowing nail | 0 (0%) | 1 (0.1%) |  | 0 (0%) |  | 1 (0.0%) |
| Inguinal hernia | 0 (0%) | 1 (0.1%) |  | 0 (0%) |  | 1 (0.0%) |
| Injection site bruising | 0 (0%) | 2 (0.2%) |  | 0 (0%) |  | 2 (0.1%) |
| Injection site haemorrhage | 0 (0%) | 3 (0.3%) |  | 0 (0%) |  | 3 (0.1%) |
| Injection site pain | 0 (0%) | 1 (0.1%) |  | 0 (0%) |  | 1 (0.0%) |
| Insomnia | 0 (0%) | 1 (0.1%) |  | 2 (0.2%) |  | 3 (0.1%) |
| Intentional device misuse | 0 (0%) | 4 (0.5%) |  | 0 (0%) |  | 4 (0.2%) |
| Intentional product misuse | 0 (0%) | 8 (0.9%) |  | 0 (0%) |  | 8 (0.3%) |
| Internal haemorrhage | 0 (0%) | 1 (0.1%) |  | 0 (0%) |  | 1 (0.0%) |
| Intervertebral disc protrusion | 0 (0%) | 2 (0.2%) |  | 1 (0.1%) |  | 3 (0.1%) |
| Irritability | 0 (0%) | 2 (0.2%) |  | 0 (0%) |  | 2 (0.1%) |
| Ischaemic cardiomyopathy | 0 (0%) | 1 (0.1%) |  | 0 (0%) |  | 1 (0.0%) |
| Kidney infection | 0 (0%) | 1 (0.1%) |  | 0 (0%) |  | 1 (0.0%) |
| Labyrinthitis | 0 (0%) | 1 (0.1%) |  | 0 (0%) |  | 1 (0.0%) |
| Laziness | 0 (0%) | 1 (0.1%) |  | 0 (0%) |  | 1 (0.0%) |
| Left ventricular dysfunction | 0 (0%) | 1 (0.1%) |  | 0 (0%) |  | 1 (0.0%) |
| Limb discomfort | 0 (0%) | 2 (0.2%) |  | 1 (0.1%) |  | 3 (0.1%) |
| Limb mass | 0 (0%) | 1 (0.1%) |  | 0 (0%) |  | 1 (0.0%) |
| Limb operation | 0 (0%) | 1 (0.1%) |  | 0 (0%) |  | 1 (0.0%) |
| Lipase increased | 0 (0%) | 1 (0.1%) |  | 0 (0%) |  | 1 (0.0%) |
| Liver contusion | 0 (0%) | 1 (0.1%) |  | 0 (0%) |  | 1 (0.0%) |
| Lung carcinoma cell type unspecified stage IV | 0 (0%) | 1 (0.1%) |  | 0 (0%) |  | 1 (0.0%) |
| Lymphadenopathy | 0 (0%) | 1 (0.1%) |  | 0 (0%) |  | 1 (0.0%) |
| Lymphoma | 0 (0%) | 1 (0.1%) |  | 1 (0.1%) |  | 2 (0.1%) |
| Mental disorder | 0 (0%) | 1 (0.1%) |  | 0 (0%) |  | 1 (0.0%) |
| Metabolic acidosis | 0 (0%) | 2 (0.2%) |  | 3 (0.3%) |  | 5 (0.2%) |
| Metabolic disorder | 0 (0%) | 1 (0.1%) |  | 0 (0%) |  | 1 (0.0%) |
| Micturition disorder | 0 (0%) | 1 (0.1%) |  | 1 (0.1%) |  | 2 (0.1%) |
| Micturition urgency | 0 (0%) | 1 (0.1%) |  | 2 (0.2%) |  | 3 (0.1%) |
| Mood altered | 0 (0%) | 1 (0.1%) |  | 0 (0%) |  | 1 (0.0%) |
| Movement disorder | 0 (0%) | 1 (0.1%) |  | 1 (0.1%) |  | 2 (0.1%) |
| Multi-organ disorder | 0 (0%) | 1 (0.1%) |  | 0 (0%) |  | 1 (0.0%) |
| Multiple fractures | 0 (0%) | 2 (0.2%) |  | 2 (0.2%) |  | 4 (0.2%) |
| Muscle atrophy | 0 (0%) | 1 (0.1%) |  | 0 (0%) |  | 1 (0.0%) |
| Muscle rupture | 0 (0%) | 1 (0.1%) |  | 0 (0%) |  | 1 (0.0%) |
| Muscle strain | 0 (0%) | 2 (0.2%) |  | 0 (0%) |  | 2 (0.1%) |
| Musculoskeletal disorder | 0 (0%) | 1 (0.1%) |  | 0 (0%) |  | 1 (0.0%) |
| Nasal injury | 0 (0%) | 1 (0.1%) |  | 0 (0%) |  | 1 (0.0%) |
| Nasopharyngitis | 0 (0%) | 1 (0.1%) |  | 3 (0.3%) |  | 4 (0.2%) |
| Neck pain | 0 (0%) | 1 (0.1%) |  | 0 (0%) |  | 1 (0.0%) |
| Nephrogenic anaemia | 0 (0%) | 1 (0.1%) |  | 0 (0%) |  | 1 (0.0%) |
| Nerve compression | 0 (0%) | 1 (0.1%) |  | 1 (0.1%) |  | 2 (0.1%) |
| Neuralgia | 0 (0%) | 1 (0.1%) |  | 0 (0%) |  | 1 (0.0%) |
| Night sweats | 0 (0%) | 1 (0.1%) |  | 0 (0%) |  | 1 (0.0%) |
| Oedema | 0 (0%) | 1 (0.1%) |  | 0 (0%) |  | 1 (0.0%) |
| Oedema peripheral | 0 (0%) | 1 (0.1%) |  | 1 (0.1%) |  | 2 (0.1%) |
| Oesophageal achalasia | 0 (0%) | 1 (0.1%) |  | 0 (0%) |  | 1 (0.0%) |
| Onychalgia | 0 (0%) | 1 (0.1%) |  | 0 (0%) |  | 1 (0.0%) |
| Optic nerve disorder | 0 (0%) | 1 (0.1%) |  | 0 (0%) |  | 1 (0.0%) |
| Oropharyngeal pain | 0 (0%) | 2 (0.2%) |  | 2 (0.2%) |  | 4 (0.2%) |
| Pain in jaw | 0 (0%) | 1 (0.1%) |  | 0 (0%) |  | 1 (0.0%) |
| Pancreatitis | 0 (0%) | 2 (0.2%) |  | 0 (0%) |  | 2 (0.1%) |
| Panic reaction | 0 (0%) | 1 (0.1%) |  | 0 (0%) |  | 1 (0.0%) |
| Paraesthesia | 0 (0%) | 2 (0.2%) |  | 1 (0.1%) |  | 3 (0.1%) |
| Paranoia | 0 (0%) | 1 (0.1%) |  | 0 (0%) |  | 1 (0.0%) |
| Penile pain | 0 (0%) | 1 (0.1%) |  | 0 (0%) |  | 1 (0.0%) |
| Penile swelling | 0 (0%) | 1 (0.1%) |  | 0 (0%) |  | 1 (0.0%) |
| Pericardial effusion | 0 (0%) | 1 (0.1%) |  | 0 (0%) |  | 1 (0.0%) |
| Pericarditis | 0 (0%) | 1 (0.1%) |  | 0 (0%) |  | 1 (0.0%) |
| Peripheral vascular disorder | 0 (0%) | 1 (0.1%) |  | 0 (0%) |  | 1 (0.0%) |
| Phimosis | 0 (0%) | 1 (0.1%) |  | 1 (0.1%) |  | 2 (0.1%) |
| Platelet count increased | 0 (0%) | 1 (0.1%) |  | 0 (0%) |  | 1 (0.0%) |
| Pleural effusion | 0 (0%) | 2 (0.2%) |  | 1 (0.1%) |  | 3 (0.1%) |
| Polyneuropathy | 0 (0%) | 1 (0.1%) |  | 0 (0%) |  | 1 (0.0%) |
| Polyuria | 0 (0%) | 2 (0.2%) |  | 1 (0.1%) |  | 3 (0.1%) |
| Poor quality sleep | 0 (0%) | 1 (0.1%) |  | 1 (0.1%) |  | 2 (0.1%) |
| Post procedural infection | 0 (0%) | 1 (0.1%) |  | 0 (0%) |  | 1 (0.0%) |
| Presyncope | 0 (0%) | 3 (0.3%) |  | 0 (0%) |  | 3 (0.1%) |
| Product dispensing error | 0 (0%) | 1 (0.1%) |  | 0 (0%) |  | 1 (0.0%) |
| Product dose omission issue | 0 (0%) | 6 (0.7%) |  | 0 (0%) |  | 6 (0.2%) |
| Product prescribing error | 0 (0%) | 1 (0.1%) |  | 0 (0%) |  | 1 (0.0%) |
| Product quality issue | 0 (0%) | 1 (0.1%) |  | 0 (0%) |  | 1 (0.0%) |
| Product use issue | 0 (0%) | 5 (0.6%) |  | 0 (0%) |  | 5 (0.2%) |
| Prostatic disorder | 0 (0%) | 1 (0.1%) |  | 0 (0%) |  | 1 (0.0%) |
| Protein total abnormal | 0 (0%) | 1 (0.1%) |  | 0 (0%) |  | 1 (0.0%) |
| Pruritus genital | 0 (0%) | 1 (0.1%) |  | 0 (0%) |  | 1 (0.0%) |
| Pseudohyponatraemia | 0 (0%) | 1 (0.1%) |  | 0 (0%) |  | 1 (0.0%) |
| Psoriasis | 0 (0%) | 1 (0.1%) |  | 0 (0%) |  | 1 (0.0%) |
| Pulmonary hypertension | 0 (0%) | 1 (0.1%) |  | 0 (0%) |  | 1 (0.0%) |
| Pyelonephritis | 0 (0%) | 1 (0.1%) |  | 0 (0%) |  | 1 (0.0%) |
| Red blood cell count increased | 0 (0%) | 1 (0.1%) |  | 0 (0%) |  | 1 (0.0%) |
| Renal impairment | 0 (0%) | 2 (0.2%) |  | 2 (0.2%) |  | 4 (0.2%) |
| Respiratory arrest | 0 (0%) | 1 (0.1%) |  | 0 (0%) |  | 1 (0.0%) |
| Respiratory distress | 0 (0%) | 1 (0.1%) |  | 1 (0.1%) |  | 2 (0.1%) |
| Restless legs syndrome | 0 (0%) | 1 (0.1%) |  | 0 (0%) |  | 1 (0.0%) |
| Sarcoidosis | 0 (0%) | 1 (0.1%) |  | 0 (0%) |  | 1 (0.0%) |
| Sciatica | 0 (0%) | 1 (0.1%) |  | 0 (0%) |  | 1 (0.0%) |
| Septic shock | 0 (0%) | 1 (0.1%) |  | 1 (0.1%) |  | 2 (0.1%) |
| Skin abrasion | 0 (0%) | 1 (0.1%) |  | 0 (0%) |  | 1 (0.0%) |
| Skin disorder | 0 (0%) | 1 (0.1%) |  | 0 (0%) |  | 1 (0.0%) |
| Skin lesion | 0 (0%) | 1 (0.1%) |  | 0 (0%) |  | 1 (0.0%) |
| Skull fracture | 0 (0%) | 1 (0.1%) |  | 0 (0%) |  | 1 (0.0%) |
| Sneezing | 0 (0%) | 1 (0.1%) |  | 0 (0%) |  | 1 (0.0%) |
| Somnolence | 0 (0%) | 1 (0.1%) |  | 5 (0.5%) |  | 6 (0.2%) |
| Spinal column injury | 0 (0%) | 2 (0.2%) |  | 0 (0%) |  | 2 (0.1%) |
| Spinal stenosis | 0 (0%) | 1 (0.1%) |  | 0 (0%) |  | 1 (0.0%) |
| Subcutaneous abscess | 0 (0%) | 1 (0.1%) |  | 0 (0%) |  | 1 (0.0%) |
| Sudden onset of sleep | 0 (0%) | 1 (0.1%) |  | 0 (0%) |  | 1 (0.0%) |
| Swelling | 0 (0%) | 2 (0.2%) |  | 1 (0.1%) |  | 3 (0.1%) |
| Swollen tongue | 0 (0%) | 1 (0.1%) |  | 0 (0%) |  | 1 (0.0%) |
| Syncope | 0 (0%) | 8 (0.9%) |  | 8 (0.8%) |  | 16 (0.6%) |
| Therapeutic response unexpected | 0 (0%) | 1 (0.1%) |  | 0 (0%) |  | 1 (0.0%) |
| Thinking abnormal | 0 (0%) | 1 (0.1%) |  | 1 (0.1%) |  | 2 (0.1%) |
| Throat irritation | 0 (0%) | 1 (0.1%) |  | 0 (0%) |  | 1 (0.0%) |
| Thrombosis | 0 (0%) | 3 (0.3%) |  | 4 (0.4%) |  | 7 (0.3%) |
| Thyroid function test abnormal | 0 (0%) | 1 (0.1%) |  | 0 (0%) |  | 1 (0.0%) |
| Tinnitus | 0 (0%) | 1 (0.1%) |  | 0 (0%) |  | 1 (0.0%) |
| Tooth disorder | 0 (0%) | 1 (0.1%) |  | 0 (0%) |  | 1 (0.0%) |
| Tooth fracture | 0 (0%) | 2 (0.2%) |  | 3 (0.3%) |  | 5 (0.2%) |
| Traumatic fracture | 0 (0%) | 1 (0.1%) |  | 0 (0%) |  | 1 (0.0%) |
| Traumatic haemorrhage | 0 (0%) | 1 (0.1%) |  | 0 (0%) |  | 1 (0.0%) |
| Type 1 diabetes mellitus | 0 (0%) | 1 (0.1%) |  | 0 (0%) |  | 1 (0.0%) |
| Underdose | 0 (0%) | 2 (0.2%) |  | 1 (0.1%) |  | 3 (0.1%) |
| Unresponsive to stimuli | 0 (0%) | 1 (0.1%) |  | 0 (0%) |  | 1 (0.0%) |
| Urethritis noninfective | 0 (0%) | 1 (0.1%) |  | 0 (0%) |  | 1 (0.0%) |
| Urinary tract disorder | 0 (0%) | 1 (0.1%) |  | 0 (0%) |  | 1 (0.0%) |
| Urinary tract obstruction | 0 (0%) | 1 (0.1%) |  | 0 (0%) |  | 1 (0.0%) |
| Vaginal discharge | 0 (0%) | 1 (0.1%) |  | 0 (0%) |  | 1 (0.0%) |
| Vaginal disorder | 0 (0%) | 1 (0.1%) |  | 0 (0%) |  | 1 (0.0%) |
| Vascular insufficiency | 0 (0%) | 1 (0.1%) |  | 0 (0%) |  | 1 (0.0%) |
| Vision blurred | 0 (0%) | 1 (0.1%) |  | 1 (0.1%) |  | 2 (0.1%) |
| Vitamin D decreased | 0 (0%) | 1 (0.1%) |  | 0 (0%) |  | 1 (0.0%) |
| Vulvovaginal pruritus | 0 (0%) | 1 (0.1%) |  | 0 (0%) |  | 1 (0.0%) |
| Weight fluctuation | 0 (0%) | 1 (0.1%) |  | 0 (0%) |  | 1 (0.0%) |
| Wrong technique in device usage process | 0 (0%) | 1 (0.1%) |  | 0 (0%) |  | 1 (0.0%) |
|  |  |  |  |  |  |  |
|  |  |  |  |  |  |  |
| Abdominal distension | 0 (0%) | 0 (0%) |  | 2 (0.2%) |  | 2 (0.1%) |
| Acute respiratory failure | 0 (0%) | 0 (0%) |  | 1 (0.1%) |  | 1 (0.0%) |
| Agitation | 0 (0%) | 0 (0%) |  | 1 (0.1%) |  | 1 (0.0%) |
| Amnesia | 0 (0%) | 0 (0%) |  | 1 (0.1%) |  | 1 (0.0%) |
| Aortic aneurysm | 0 (0%) | 0 (0%) |  | 2 (0.2%) |  | 2 (0.1%) |
| Aortic valve disease | 0 (0%) | 0 (0%) |  | 1 (0.1%) |  | 1 (0.0%) |
| Aphonia | 0 (0%) | 0 (0%) |  | 1 (0.1%) |  | 1 (0.0%) |
| Bacterial infection | 0 (0%) | 0 (0%) |  | 1 (0.1%) |  | 1 (0.0%) |
| Bacterial pyelonephritis | 0 (0%) | 0 (0%) |  | 1 (0.1%) |  | 1 (0.0%) |
| Base excess decreased | 0 (0%) | 0 (0%) |  | 1 (0.1%) |  | 1 (0.0%) |
| Bedridden | 0 (0%) | 0 (0%) |  | 3 (0.3%) |  | 3 (0.1%) |
| Benign prostatic hyperplasia | 0 (0%) | 0 (0%) |  | 1 (0.1%) |  | 1 (0.0%) |
| Bladder disorder | 0 (0%) | 0 (0%) |  | 2 (0.2%) |  | 2 (0.1%) |
| Bladder irritation | 0 (0%) | 0 (0%) |  | 1 (0.1%) |  | 1 (0.0%) |
| Blood bicarbonate decreased | 0 (0%) | 0 (0%) |  | 1 (0.1%) |  | 1 (0.0%) |
| Blood creatine increased | 0 (0%) | 0 (0%) |  | 1 (0.1%) |  | 1 (0.0%) |
| Blood electrolytes abnormal | 0 (0%) | 0 (0%) |  | 1 (0.1%) |  | 1 (0.0%) |
| Blood glucose fluctuation | 0 (0%) | 0 (0%) |  | 2 (0.2%) |  | 2 (0.1%) |
| Blood ketone body present | 0 (0%) | 0 (0%) |  | 1 (0.1%) |  | 1 (0.0%) |
| Blood lactic acid increased | 0 (0%) | 0 (0%) |  | 1 (0.1%) |  | 1 (0.0%) |
| Blood pH increased | 0 (0%) | 0 (0%) |  | 1 (0.1%) |  | 1 (0.0%) |
| Blood pressure diastolic decreased | 0 (0%) | 0 (0%) |  | 1 (0.1%) |  | 1 (0.0%) |
| Blood pressure diastolic increased | 0 (0%) | 0 (0%) |  | 1 (0.1%) |  | 1 (0.0%) |
| Blood pressure fluctuation | 0 (0%) | 0 (0%) |  | 1 (0.1%) |  | 1 (0.0%) |
| Blood pressure systolic abnormal | 0 (0%) | 0 (0%) |  | 1 (0.1%) |  | 1 (0.0%) |
| Blood pressure systolic increased | 0 (0%) | 0 (0%) |  | 1 (0.1%) |  | 1 (0.0%) |
| Blood sodium decreased | 0 (0%) | 0 (0%) |  | 1 (0.1%) |  | 1 (0.0%) |
| Blood thyroid stimulating hormone increased | 0 (0%) | 0 (0%) |  | 1 (0.1%) |  | 1 (0.0%) |
| Brain contusion | 0 (0%) | 0 (0%) |  | 1 (0.1%) |  | 1 (0.0%) |
| Breast cancer | 0 (0%) | 0 (0%) |  | 1 (0.1%) |  | 1 (0.0%) |
| Breast pain | 0 (0%) | 0 (0%) |  | 1 (0.1%) |  | 1 (0.0%) |
| Bursa removal | 0 (0%) | 0 (0%) |  | 1 (0.1%) |  | 1 (0.0%) |
| C-reactive protein increased | 0 (0%) | 0 (0%) |  | 1 (0.1%) |  | 1 (0.0%) |
| Candida infection | 0 (0%) | 0 (0%) |  | 1 (0.1%) |  | 1 (0.0%) |
| Carbohydrate metabolism disorder | 0 (0%) | 0 (0%) |  | 1 (0.1%) |  | 1 (0.0%) |
| Carbon dioxide increased | 0 (0%) | 0 (0%) |  | 1 (0.1%) |  | 1 (0.0%) |
| Cardiac aneurysm | 0 (0%) | 0 (0%) |  | 1 (0.1%) |  | 1 (0.0%) |
| Cardiac discomfort | 0 (0%) | 0 (0%) |  | 1 (0.1%) |  | 1 (0.0%) |
| Cardiac valve disease | 0 (0%) | 0 (0%) |  | 1 (0.1%) |  | 1 (0.0%) |
| Cerebral thrombosis | 0 (0%) | 0 (0%) |  | 1 (0.1%) |  | 1 (0.0%) |
| Cervical vertebral fracture | 0 (0%) | 0 (0%) |  | 1 (0.1%) |  | 1 (0.0%) |
| Cholecystitis | 0 (0%) | 0 (0%) |  | 1 (0.1%) |  | 1 (0.0%) |
| Cholelithiasis | 0 (0%) | 0 (0%) |  | 1 (0.1%) |  | 1 (0.0%) |
| Circulatory collapse | 0 (0%) | 0 (0%) |  | 1 (0.1%) |  | 1 (0.0%) |
| Coccydynia | 0 (0%) | 0 (0%) |  | 1 (0.1%) |  | 1 (0.0%) |
| Colonoscopy | 0 (0%) | 0 (0%) |  | 1 (0.1%) |  | 1 (0.0%) |
| Coronary arterial stent insertion | 0 (0%) | 0 (0%) |  | 1 (0.1%) |  | 1 (0.0%) |
| Cough | 0 (0%) | 0 (0%) |  | 1 (0.1%) |  | 1 (0.0%) |
| COVID-19 | 0 (0%) | 0 (0%) |  | 5 (0.5%) |  | 5 (0.2%) |
| COVID-19 pneumonia | 0 (0%) | 0 (0%) |  | 2 (0.2%) |  | 2 (0.1%) |
| Cyst | 0 (0%) | 0 (0%) |  | 1 (0.1%) |  | 1 (0.0%) |
| Decubitus ulcer | 0 (0%) | 0 (0%) |  | 1 (0.1%) |  | 1 (0.0%) |
| Dementia | 0 (0%) | 0 (0%) |  | 1 (0.1%) |  | 1 (0.0%) |
| Dental caries | 0 (0%) | 0 (0%) |  | 1 (0.1%) |  | 1 (0.0%) |
| Depression | 0 (0%) | 0 (0%) |  | 1 (0.1%) |  | 1 (0.0%) |
| Device physical property issue | 0 (0%) | 0 (0%) |  | 1 (0.1%) |  | 1 (0.0%) |
| Diabetic nephropathy | 0 (0%) | 0 (0%) |  | 1 (0.1%) |  | 1 (0.0%) |
| Dialysis | 0 (0%) | 0 (0%) |  | 2 (0.2%) |  | 2 (0.1%) |
| Disability | 0 (0%) | 0 (0%) |  | 1 (0.1%) |  | 1 (0.0%) |
| Diverticulum | 0 (0%) | 0 (0%) |  | 1 (0.1%) |  | 1 (0.0%) |
| Drug dispensed to wrong patient | 0 (0%) | 0 (0%) |  | 1 (0.1%) |  | 1 (0.0%) |
| Drug eruption | 0 (0%) | 0 (0%) |  | 1 (0.1%) |  | 1 (0.0%) |
| Drug intolerance | 0 (0%) | 0 (0%) |  | 1 (0.1%) |  | 1 (0.0%) |
| Drug titration error | 0 (0%) | 0 (0%) |  | 1 (0.1%) |  | 1 (0.0%) |
| Dry throat | 0 (0%) | 0 (0%) |  | 1 (0.1%) |  | 1 (0.0%) |
| Dyscalculia | 0 (0%) | 0 (0%) |  | 1 (0.1%) |  | 1 (0.0%) |
| Dysgeusia | 0 (0%) | 0 (0%) |  | 1 (0.1%) |  | 1 (0.0%) |
| Ear infection | 0 (0%) | 0 (0%) |  | 1 (0.1%) |  | 1 (0.0%) |
| Electrolyte imbalance | 0 (0%) | 0 (0%) |  | 2 (0.2%) |  | 2 (0.1%) |
| Enzyme level increased | 0 (0%) | 0 (0%) |  | 1 (0.1%) |  | 1 (0.0%) |
| Erythema | 0 (0%) | 0 (0%) |  | 1 (0.1%) |  | 1 (0.0%) |
| Feeling cold | 0 (0%) | 0 (0%) |  | 1 (0.1%) |  | 1 (0.0%) |
| Feeling hot | 0 (0%) | 0 (0%) |  | 2 (0.2%) |  | 2 (0.1%) |
| Flushing | 0 (0%) | 0 (0%) |  | 1 (0.1%) |  | 1 (0.0%) |
| Fractured coccyx | 0 (0%) | 0 (0%) |  | 1 (0.1%) |  | 1 (0.0%) |
| Gallbladder polyp | 0 (0%) | 0 (0%) |  | 1 (0.1%) |  | 1 (0.0%) |
| Gastroenteritis viral | 0 (0%) | 0 (0%) |  | 1 (0.1%) |  | 1 (0.0%) |
| Gastrointestinal disorder | 0 (0%) | 0 (0%) |  | 1 (0.1%) |  | 1 (0.0%) |
| Gastrointestinal pain | 0 (0%) | 0 (0%) |  | 1 (0.1%) |  | 1 (0.0%) |
| Genital infection male | 0 (0%) | 0 (0%) |  | 1 (0.1%) |  | 1 (0.0%) |
| Glomerular filtration rate abnormal | 0 (0%) | 0 (0%) |  | 1 (0.1%) |  | 1 (0.0%) |
| Glomerular filtration rate decreased | 0 (0%) | 0 (0%) |  | 1 (0.1%) |  | 1 (0.0%) |
| Glomerular filtration rate increased | 0 (0%) | 0 (0%) |  | 1 (0.1%) |  | 1 (0.0%) |
| Haematocrit decreased | 0 (0%) | 0 (0%) |  | 1 (0.1%) |  | 1 (0.0%) |
| Haemoglobin decreased | 0 (0%) | 0 (0%) |  | 1 (0.1%) |  | 1 (0.0%) |
| Head discomfort | 0 (0%) | 0 (0%) |  | 1 (0.1%) |  | 1 (0.0%) |
| Heart valve replacement | 0 (0%) | 0 (0%) |  | 1 (0.1%) |  | 1 (0.0%) |
| Hepatic steatosis | 0 (0%) | 0 (0%) |  | 1 (0.1%) |  | 1 (0.0%) |
| Hip arthroplasty | 0 (0%) | 0 (0%) |  | 1 (0.1%) |  | 1 (0.0%) |
| Hyperpyrexia | 0 (0%) | 0 (0%) |  | 1 (0.1%) |  | 1 (0.0%) |
| Hypoglycaemic seizure | 0 (0%) | 0 (0%) |  | 1 (0.1%) |  | 1 (0.0%) |
| Hypomagnesaemia | 0 (0%) | 0 (0%) |  | 2 (0.2%) |  | 2 (0.1%) |
| Hypovolaemia | 0 (0%) | 0 (0%) |  | 1 (0.1%) |  | 1 (0.0%) |
| Impaired quality of life | 0 (0%) | 0 (0%) |  | 1 (0.1%) |  | 1 (0.0%) |
| Incorrect product formulation administered | 0 (0%) | 0 (0%) |  | 1 (0.1%) |  | 1 (0.0%) |
| Inflammatory marker increased | 0 (0%) | 0 (0%) |  | 1 (0.1%) |  | 1 (0.0%) |
| Inner ear disorder | 0 (0%) | 0 (0%) |  | 1 (0.1%) |  | 1 (0.0%) |
| Intracranial pressure increased | 0 (0%) | 0 (0%) |  | 1 (0.1%) |  | 1 (0.0%) |
| Joint swelling | 0 (0%) | 0 (0%) |  | 1 (0.1%) |  | 1 (0.0%) |
| Ketosis | 0 (0%) | 0 (0%) |  | 1 (0.1%) |  | 1 (0.0%) |
| Knee arthroplasty | 0 (0%) | 0 (0%) |  | 2 (0.2%) |  | 2 (0.1%) |
| Knee operation | 0 (0%) | 0 (0%) |  | 1 (0.1%) |  | 1 (0.0%) |
| Lactic acidosis | 0 (0%) | 0 (0%) |  | 2 (0.2%) |  | 2 (0.1%) |
| Ligament rupture | 0 (0%) | 0 (0%) |  | 1 (0.1%) |  | 1 (0.0%) |
| Lip dry | 0 (0%) | 0 (0%) |  | 1 (0.1%) |  | 1 (0.0%) |
| Lip exfoliation | 0 (0%) | 0 (0%) |  | 2 (0.2%) |  | 2 (0.1%) |
| Lip swelling | 0 (0%) | 0 (0%) |  | 1 (0.1%) |  | 1 (0.0%) |
| Lumbar vertebral fracture | 0 (0%) | 0 (0%) |  | 3 (0.3%) |  | 3 (0.1%) |
| Lung disorder | 0 (0%) | 0 (0%) |  | 2 (0.2%) |  | 2 (0.1%) |
| Lung neoplasm | 0 (0%) | 0 (0%) |  | 1 (0.1%) |  | 1 (0.0%) |
| Lymphocyte count decreased | 0 (0%) | 0 (0%) |  | 1 (0.1%) |  | 1 (0.0%) |
| Medical device implantation | 0 (0%) | 0 (0%) |  | 1 (0.1%) |  | 1 (0.0%) |
| Melaena | 0 (0%) | 0 (0%) |  | 1 (0.1%) |  | 1 (0.0%) |
| Memory impairment | 0 (0%) | 0 (0%) |  | 2 (0.2%) |  | 2 (0.1%) |
| Meningitis aseptic | 0 (0%) | 0 (0%) |  | 1 (0.1%) |  | 1 (0.0%) |
| Metabolic encephalopathy | 0 (0%) | 0 (0%) |  | 1 (0.1%) |  | 1 (0.0%) |
| Migraine | 0 (0%) | 0 (0%) |  | 1 (0.1%) |  | 1 (0.0%) |
| Mucosal disorder | 0 (0%) | 0 (0%) |  | 1 (0.1%) |  | 1 (0.0%) |
| Multiple organ dysfunction syndrome | 0 (0%) | 0 (0%) |  | 1 (0.1%) |  | 1 (0.0%) |
| Musculoskeletal discomfort | 0 (0%) | 0 (0%) |  | 1 (0.1%) |  | 1 (0.0%) |
| Musculoskeletal stiffness | 0 (0%) | 0 (0%) |  | 1 (0.1%) |  | 1 (0.0%) |
| Myalgia | 0 (0%) | 0 (0%) |  | 2 (0.2%) |  | 2 (0.1%) |
| N-terminal prohormone brain natriuretic peptide increased | 0 (0%) | 0 (0%) |  | 1 (0.1%) |  | 1 (0.0%) |
| Nail disorder | 0 (0%) | 0 (0%) |  | 1 (0.1%) |  | 1 (0.0%) |
| Neoplasm | 0 (0%) | 0 (0%) |  | 1 (0.1%) |  | 1 (0.0%) |
| Neoplasm malignant | 0 (0%) | 0 (0%) |  | 1 (0.1%) |  | 1 (0.0%) |
| Nephrolithiasis | 0 (0%) | 0 (0%) |  | 1 (0.1%) |  | 1 (0.0%) |
| Nephropathy | 0 (0%) | 0 (0%) |  | 1 (0.1%) |  | 1 (0.0%) |
| Obstructive sleep apnoea syndrome | 0 (0%) | 0 (0%) |  | 1 (0.1%) |  | 1 (0.0%) |
| Oesophageal food impaction | 0 (0%) | 0 (0%) |  | 1 (0.1%) |  | 1 (0.0%) |
| Otitis media | 0 (0%) | 0 (0%) |  | 1 (0.1%) |  | 1 (0.0%) |
| Oxygen saturation decreased | 0 (0%) | 0 (0%) |  | 2 (0.2%) |  | 2 (0.1%) |
| Oxygen saturation increased | 0 (0%) | 0 (0%) |  | 1 (0.1%) |  | 1 (0.0%) |
| Pancreatic carcinoma | 0 (0%) | 0 (0%) |  | 1 (0.1%) |  | 1 (0.0%) |
| Parathyroid disorder | 0 (0%) | 0 (0%) |  | 1 (0.1%) |  | 1 (0.0%) |
| Pelvic fracture | 0 (0%) | 0 (0%) |  | 1 (0.1%) |  | 1 (0.0%) |
| Pelvic pain | 0 (0%) | 0 (0%) |  | 2 (0.2%) |  | 2 (0.1%) |
| Penis disorder | 0 (0%) | 0 (0%) |  | 1 (0.1%) |  | 1 (0.0%) |
| Peripheral artery occlusion | 0 (0%) | 0 (0%) |  | 1 (0.1%) |  | 1 (0.0%) |
| Pharyngeal swelling | 0 (0%) | 0 (0%) |  | 1 (0.1%) |  | 1 (0.0%) |
| Pharyngitis streptococcal | 0 (0%) | 0 (0%) |  | 1 (0.1%) |  | 1 (0.0%) |
| Pituitary tumour benign | 0 (0%) | 0 (0%) |  | 1 (0.1%) |  | 1 (0.0%) |
| Plasma cell myeloma | 0 (0%) | 0 (0%) |  | 1 (0.1%) |  | 1 (0.0%) |
| Polydipsia | 0 (0%) | 0 (0%) |  | 1 (0.1%) |  | 1 (0.0%) |
| Poor venous access | 0 (0%) | 0 (0%) |  | 1 (0.1%) |  | 1 (0.0%) |
| Product dose omission in error | 0 (0%) | 0 (0%) |  | 1 (0.1%) |  | 1 (0.0%) |
| Pulmonary oedema | 0 (0%) | 0 (0%) |  | 1 (0.1%) |  | 1 (0.0%) |
| Pulmonary thrombosis | 0 (0%) | 0 (0%) |  | 1 (0.1%) |  | 1 (0.0%) |
| Pulse pressure increased | 0 (0%) | 0 (0%) |  | 1 (0.1%) |  | 1 (0.0%) |
| Rash vesicular | 0 (0%) | 0 (0%) |  | 1 (0.1%) |  | 1 (0.0%) |
| Rectal haemorrhage | 0 (0%) | 0 (0%) |  | 1 (0.1%) |  | 1 (0.0%) |
| Red blood cell count decreased | 0 (0%) | 0 (0%) |  | 1 (0.1%) |  | 1 (0.0%) |
| Renal cyst | 0 (0%) | 0 (0%) |  | 1 (0.1%) |  | 1 (0.0%) |
| Renal disorder | 0 (0%) | 0 (0%) |  | 2 (0.2%) |  | 2 (0.1%) |
| Respiratory rate increased | 0 (0%) | 0 (0%) |  | 2 (0.2%) |  | 2 (0.1%) |
| Respiratory tract congestion | 0 (0%) | 0 (0%) |  | 1 (0.1%) |  | 1 (0.0%) |
| Restlessness | 0 (0%) | 0 (0%) |  | 1 (0.1%) |  | 1 (0.0%) |
| Rheumatoid arthritis | 0 (0%) | 0 (0%) |  | 1 (0.1%) |  | 1 (0.0%) |
| Rhinorrhoea | 0 (0%) | 0 (0%) |  | 1 (0.1%) |  | 1 (0.0%) |
| Sarcoma of skin | 0 (0%) | 0 (0%) |  | 1 (0.1%) |  | 1 (0.0%) |
| Scrotal pain | 0 (0%) | 0 (0%) |  | 1 (0.1%) |  | 1 (0.0%) |
| Scrotal ulcer | 0 (0%) | 0 (0%) |  | 1 (0.1%) |  | 1 (0.0%) |
| Seasonal allergy | 0 (0%) | 0 (0%) |  | 1 (0.1%) |  | 1 (0.0%) |
| Sinusitis | 0 (0%) | 0 (0%) |  | 1 (0.1%) |  | 1 (0.0%) |
| Skin discomfort | 0 (0%) | 0 (0%) |  | 1 (0.1%) |  | 1 (0.0%) |
| Skin injury | 0 (0%) | 0 (0%) |  | 1 (0.1%) |  | 1 (0.0%) |
| Skin odour abnormal | 0 (0%) | 0 (0%) |  | 1 (0.1%) |  | 1 (0.0%) |
| Skull fractured base | 0 (0%) | 0 (0%) |  | 1 (0.1%) |  | 1 (0.0%) |
| Speech disorder | 0 (0%) | 0 (0%) |  | 1 (0.1%) |  | 1 (0.0%) |
| Staphylococcal abscess | 0 (0%) | 0 (0%) |  | 1 (0.1%) |  | 1 (0.0%) |
| Starvation | 0 (0%) | 0 (0%) |  | 1 (0.1%) |  | 1 (0.0%) |
| Sternal fracture | 0 (0%) | 0 (0%) |  | 1 (0.1%) |  | 1 (0.0%) |
| Stress | 0 (0%) | 0 (0%) |  | 1 (0.1%) |  | 1 (0.0%) |
| Subarachnoid haemorrhage | 0 (0%) | 0 (0%) |  | 3 (0.3%) |  | 3 (0.1%) |
| Subdural haematoma | 0 (0%) | 0 (0%) |  | 2 (0.2%) |  | 2 (0.1%) |
| Subdural haemorrhage | 0 (0%) | 0 (0%) |  | 1 (0.1%) |  | 1 (0.0%) |
| Surgery | 0 (0%) | 0 (0%) |  | 3 (0.3%) |  | 3 (0.1%) |
| Swelling face | 0 (0%) | 0 (0%) |  | 1 (0.1%) |  | 1 (0.0%) |
| Tachypnoea | 0 (0%) | 0 (0%) |  | 1 (0.1%) |  | 1 (0.0%) |
| Therapy interrupted | 0 (0%) | 0 (0%) |  | 1 (0.1%) |  | 1 (0.0%) |
| Thoracic vertebral fracture | 0 (0%) | 0 (0%) |  | 1 (0.1%) |  | 1 (0.0%) |
| Thyroid mass | 0 (0%) | 0 (0%) |  | 1 (0.1%) |  | 1 (0.0%) |
| Tooth abscess | 0 (0%) | 0 (0%) |  | 1 (0.1%) |  | 1 (0.0%) |
| Tooth erosion | 0 (0%) | 0 (0%) |  | 1 (0.1%) |  | 1 (0.0%) |
| Toothache | 0 (0%) | 0 (0%) |  | 1 (0.1%) |  | 1 (0.0%) |
| Treatment failure | 0 (0%) | 0 (0%) |  | 1 (0.1%) |  | 1 (0.0%) |
| Ulna fracture | 0 (0%) | 0 (0%) |  | 1 (0.1%) |  | 1 (0.0%) |
| Urine abnormality | 0 (0%) | 0 (0%) |  | 1 (0.1%) |  | 1 (0.0%) |
| Urine ketone body | 0 (0%) | 0 (0%) |  | 1 (0.1%) |  | 1 (0.0%) |
| Urine odour abnormal | 0 (0%) | 0 (0%) |  | 1 (0.1%) |  | 1 (0.0%) |
| White blood cell disorder | 0 (0%) | 0 (0%) |  | 1 (0.1%) |  | 1 (0.0%) |

**According to Medical Dictionary for Regulatory Activities (MedDRA) version 24.1.*

**Table S3**. Fall and fractures listed in Individual Case Safety Reports (ICSRs) with DPP4 inhibitors and reported in Eudravigilance from January 1st. 2015 to December 31st. 2022.

|  | **DPP4 INHIBITORS (N=513)** |
| --- | --- |
| **Preferred terms*** |  |
| Acetabulum fracture | 1 (0.2%) |
| Ankle fracture | 10 (1.9%) |
| Atypical femur fracture | 1 (0.2%) |
| Cervical vertebral fracture | 3 (0.6%) |
| Clavicle fracture | 1 (0.2%) |
| Compression fracture | 3 (0.6%) |
| Fall | 318 (62.0%) |
| Femoral neck fracture | 15 (2.9%) |
| Femur fracture | 21 (4.1%) |
| Fibula fracture | 1 (0.2%) |
| Foot fracture | 4 (0.8%) |
| Forearm fracture | 1 (0.2%) |
| Fracture | 23 (4.5%) |
| Fractured sacrum | 1 (0.2%) |
| Hand fracture | 3 (0.6%) |
| Hip fracture | 21 (4.1%) |
| Humerus fracture | 3 (0.6%) |
| Jaw fracture | 2 (0.4%) |
| Lower limb fracture | 14 (2.7%) |
| Lumbar vertebral fracture | 3 (0.6%) |
| Multiple fractures | 3 (0.6%) |
| Open fracture | 1 (0.2%) |
| Patella fracture | 1 (0.2%) |
| Pelvic fracture | 5 (1.0%) |
| Radius fracture | 3 (0.6%) |
| Rib fracture | 12 (2.3%) |
| Spinal compression fracture | 8 (1.6%) |
| Spinal fracture | 5 (1.0%) |
| Thoracic vertebral fracture | 1 (0.2%) |
| Tibia fracture | 1 (0.2%) |
| Tooth fracture | 1 (0.2%) |
| Traumatic fracture | 1 (0.2%) |
| Upper limb fracture | 19 (3.7%) |
| Wrist fracture | 3 (0.6%) |

**According to Medical Dictionary for Regulatory Activities (MedDRA) version 24.1.*

**Table S4.** Reporting probabilities of fractures between SGLT2 inhibitors and DPP4 inhibitors excluding cases with the presence of both pharmacological treatments expressed as Reporting Odds Ratios (RORs) and their 95% Confidence Intervals (95%CI).

| Comparison | ROR | 95% Confidence Interval | P-Value |
| --- | --- | --- | --- |
| *Dapagliflozin vs. DPP4 inhibitors* | 0.80 | 0.60-1.06 | 0.1177 |
| *Canagliflozin vs. DPP4 inhibitors* | 1.14 | 0.90-1.43 | 0.2893 |
| *Empagliflozin vs. DPP4 inhibitors* | 0.94 | 0.73-1.22 | 0.6472 |
| *SGLT2 inhibitors vs. DPP4 inhibitors* | 0.97 | 0.80-1.17 | 0.7354 |

**Table S5.** Reporting probabilities of fall between SGLT2 inhibitors and DPP4 inhibitors excluding cases with the presence of both pharmacological treatments expressed as Reporting Odds Ratios (RORs) and their 95% Confidence Intervals (95%CI).

| Comparison | ROR | 95% Confidence Interval | P-Value |
| --- | --- | --- | --- |
| *Dapagliflozin vs. DPP4 inhibitors* | 0.57 | 0.44-0.73 | 0.0000 |
| *Canagliflozin vs. DPP4 inhibitors* | 0.55 | 0.44-0.70 | 0.0000 |
| *Empagliflozin vs. DPP4 inhibitors* | 0.78 | 0.63-0.97 | 0.0230 |
| *SGLT2 inhibitors vs. DPP4 inhibitors* | 0.64 | 0.54-0.75 | 0.0000 |
